# Ripples in Graphene: A Variational Approach

**DOI:** 10.1007/s00220-020-03869-z

**Published:** 2020-10-06

**Authors:** Manuel Friedrich, Ulisse Stefanelli

**Affiliations:** 1 Applied Mathematics, Universität Münster, Einsteinstr. 62, 48149 Münster, Germany; 2 Faculty of Mathematics, University of Vienna, Oskar-Morgenstern-Platz 1, 1090 Vienna, Austria; 3 Vienna Research Platform on Accelerating Photoreaction Discovery, University of Vienna, Währinger Str. 17, 1090 Vienna, Austria; 4 Istituto di Matematica Applicata e Tecnologie Informatiche “E. Magenes” - CNR, v. Ferrata 1, 27100 Pavia, Italy

## Abstract

Suspended graphene samples are observed to be gently rippled rather than being flat. In Friedrich et al. (Z Angew Math Phys 69:70, 2018), we have checked that this nonplanarity can be rigorously described within the classical molecular-mechanical frame of configurational-energy minimization. There, we have identified all ground-state configurations with graphene topology with respect to classes of next-to-nearest neighbor interaction energies and classified their fine nonflat geometries. In this second paper on graphene nonflatness, we refine the analysis further and prove the emergence of wave patterning. Moving within the frame of Friedrich et al. (2018), rippling formation in graphene is reduced to a two-dimensional problem for one-dimensional chains. Specifically, we show that almost minimizers of the configurational energy develop waves with specific wavelength, independently of the size of the sample. This corresponds remarkably to experiments and simulations.

## Introduction

Carbon forms a variety of different allotropic nanostructures. Among these a prominent role is played by graphene, a pure-carbon structure consisting of a one-atom thick layer of atoms arranged in a hexagonal lattice. Its serendipitous discovery in 2005 has been awarded the 2010 Nobel Prize in Physics to Geim and Novoselov and has sparkled an exponentially growing research activity. The fascinating electronic properties of graphene are believed to offer unprecedented opportunities for innovative applications, ranging from next-generation electronics to pharmacology, and including batteries and solar cells. A new branch of Materials Science dedicated to lower-dimensional systems has developed, cutting across Physics and Chemistry and extending from fundamental science to production [8].

Despite the progressive growth of experimental, computational, and theoretical understanding of graphene, the accurate description of its fine geometry remains to date still elusive. Indeed, suspended graphene samples are not exactly flat but gently rippled [1, 22] and waves of approximately one hundred atom spacings have been predicted computationally [7]. Such departure from planarity seems to be necessary in order to achieve stability at finite temperatures, in accordance with the limitations imposed by the classical Mermin–Wagner Theorem [15, 20, 21]. Even in the zero-temperature limit, recent computations [12] suggest that nonplanarity is still be expected due to quantum fluctuations. Note that, beside the academic interest, the fine geometry of graphene sheets is of a great applicative importance, for it is considered to be the relevant scattering mechanism limiting electronic mobility [13, 26].

The aim of this paper is to prove that the emergence of waves with a specific, sample-size independent wavelength can be rigorously predicted. We move within the frame of Molecular Mechanics, which consists in describing the carbon atoms as classical particles and in investigating minimality with respect to a corresponding configurational energy. This energy is given in terms of classical potentials and takes into account both attractive-repulsive *two-body* interactions, minimized at some given bond length, and *three-body* terms favoring specific angles between bonds [2, 3, 24, 25]. With respect to quantum-mechanical models, Molecular Mechanics has the advantage of being simpler and parametrizable, although at the expense of a certain degree of approximation. Remarkably, it delivers the only computationally amenable option as the size of the system scales up. In addition, it often allows for a rigorous mathematical analysis. In particular, crystallization results for graphene in two dimensions have been proved both in the thermodynamic limit setting [5, 6] and in the case of a finite number of atoms [4, 19]. The fine geometry of other carbon nanostructures has also been investigated [9, 10, 16–18, 23].

**Fig. 1:**
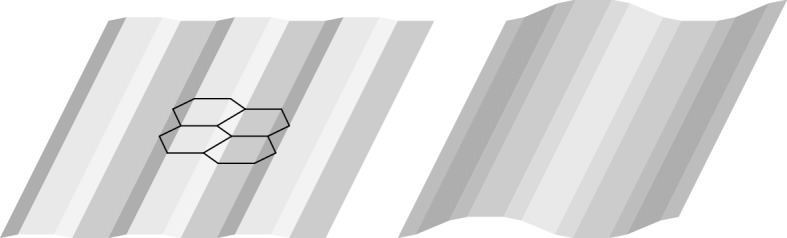
Examples of rippled structures

A first step toward the understanding of rippling in graphene is detailed in the companion paper [11] where we investigate ground-state deformations of the regular hexagonal lattice with respect to configurational energies including next-to-nearest-neighbor interactions. (Note that pure nearest-neighbor interactions predict flat minimizers.) In such setting, optimal hexagonal cells are not planar, see Fig. 1 left. The main result of [11] is a classification of all graphene ground states into two distinct families: rolled-up and rippled configurations. Rolled-up structures ideally correspond to carbon nanotubes. Their optimality recalls remarkably the experimental evidence that free-standing graphene samples tend to roll up [14]. Rippled configurations, see Fig. 1, would instead correspond to *suspended* graphene patches, where the rolling-up is prevented by the adhesion to a probing frame.

Our focus is here on rippled configurations. These are not planar and feature a specific direction in three-dimensional space along which they are periodic. The full three-dimensional description of rippled configurations is hence completely determined by orthogonal sections to such specific periodicity direction ( see the free edge at the bottom of the samples in Fig. 1, for instance). The aim of this paper is to address the geometry of such orthogonal sections (and hence of the whole rippled configuration) from a variational viewpoint. In fact, such sections are nothing but one-dimensional chains in two dimensions.

**Fig. 2:**
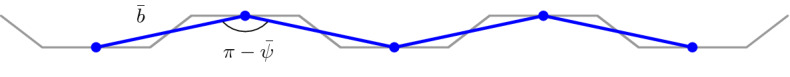
Effective description of the section of the rippled structure on the right of Fig. 1

We introduce an *effective energy* for such sections by considering cell centers as particles and favoring a specific distance ${\bar{b}}$ between cell centers and a specific angle $\pi - {\bar{\psi }}$ between segments connecting neighboring cell centers. Figure 2 illustrates this setting in the case of the rippled configuration on the left of Fig. 1.

Specific wave patterns of the rippled structure will then correspond to waves in the chain of cell centers, as in the case of the rippled configuration on the right of Fig. 1. By slightly abusing terminology, we shall hence call *particles* such cell centers and *bonds* the segments between two neighboring cell centers.

**Fig. 3:**
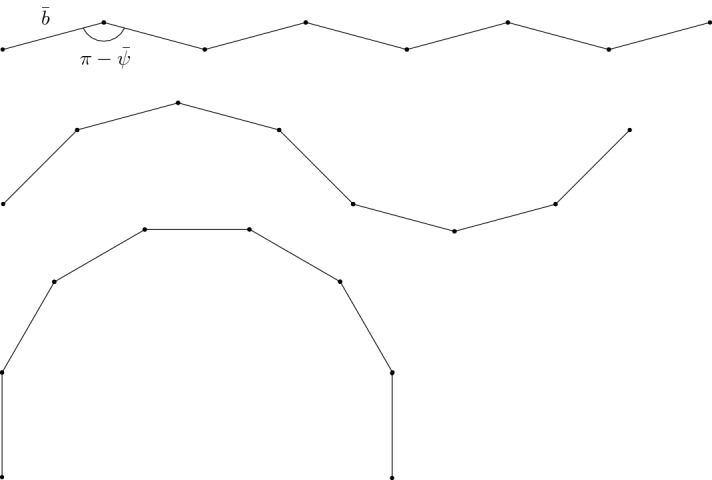
Energy minimizers of (3) with different geometries

In the following, two choices for the effective energy are considered. At first, we analyze the *reduced energy* (3) taking into account nearest- and next-to-nearest-neighbor interactions and favoring nonaligned consecutive bonds. This leads to a large variety of energy minimizers with many different geometries, see Fig. 3. We then specialize the description via the *(general) energy* (7) taking additionally longer-range interactions into account. This second choice leads to a finer characterization of energy minimizers since the energy accounts also for curvature changes of the chain.

Our main result (Theorem 2.4) states the possibility of finding an optimal wavelength for energy minimizers. More precisely, for all prescribed overall lengths of the chain one finds an optimal wavelength $\lambda$ such that all almost minimizers of the energy with that specific length can be viewed as compositions of $\lambda$ waves, up to lower-order terms. Note that by fixing a given length of the chain one actually imposes a boundary condition, which corresponds to suspending the sample. Without such a boundary condition, no optimal wavelength is to be expected, for the sample would be rolling up, an instance which is indeed captured by our description. The crucial point of our result is that the optimal wavelength $\lambda$ is independent of the size of the system. This corresponds to experimental and computational findings [7, 22]. It is worth at this point to emphasize that the model features no ad-hoc addition of a mesoscopic lengthscale and that the optimal wavelength exclusively arises from minimality.

All results of the paper are presented in Sect. 2. The corresponding proofs are based on elementary arguments but are technically very involved and are detailed in Sects. 3–6. A first step is achieved in Sect. 3 where we consider a *cell energy* depending just on three consecutive particles. Here, convexity allows to check that minimizers are configurations where the two bonds between the particles are not aligned.

In Sect. 4 we consider the *single-period problem* of a chain which changes its curvature only once. In particular, we identify the optimal *wavelength* $\lambda ^l_{\mathrm{max}}$ depending on the number of bonds *l* (later referred to as * discrete-wave period*). To this aim, it is instrumental to check for the concavity of the mappings $l \mapsto \lambda ^l_{\mathrm{max}}$ and $l \mapsto \lambda ^l_{\mathrm{max}}/l$ (see Lemma 4.3 and Lemma 4.5) where $\lambda ^l_{\mathrm{max}}/l$ represents the *normalized wavelength*. Eventually, by convexity arguments we are able to control the deviation of the length of the chain from the optimal wavelength $\lambda ^l_{\mathrm{max}}$ in terms of the energy excess, see Lemma 4.9. The strategy is then to identify candidate minimizers by composing more single-period chains, see Fig. 8 for an illustration. This turns out to be properly doable for even discrete-wave periods only. The treatment of odd discrete-wave periods is surprisingly much more intricate, see e.g. Lemma 4.8. One resorts there in showing that the combination of two single-period waves with odd discrete-wave periods is unfavored with respect to the combination of two single-period waves with even discrete-wave periods having the same overall length.

Once the single-period problem is settled, we tackle in Sect. 5 the *multiple-period problem*, by allowing the chain to change curvature more than once. We show here that the energy of the chain depends on the number of particles where the chain changes its curvature, see Lemma 5.3. We also quantify the length of the chain in terms of the number of different discrete-wave periods composing it (Lemma 5.1) and we show that the length excess can be controlled in terms of the energy excess (Lemma 5.2).

Section 6 finally contains the proof of the main result. We firstly address the characterization of the minimal energy (Theorem 2.2). The upper bound for the minimal energy is obtained via an explicit construction composing single-period chains. The proof of the matching lower bound is more subtle and relies on the fine geometry of almost minimizers. In particular, we show that a chain with almost minimal energy essentially consists exclusively of single-period chains with a specific discrete-wave period, which only depends on the choice of the boundary conditions. The main underlying observation is made in terms of normalized wavelengths (wavelength divided by discrete-wave period): (1) the normalized wavelength of chains with larger discrete-wave periods is too short to accommodate the boundary conditions and (2) chains with smaller discrete-wave period, although having sufficiently large normalized wavelength, cost too much energy due to a large number of curvature changes. The arguments rely on a fine interplay of the longer-range contributions and the wavelength $\lambda ^l_{\mathrm{max}}$ for different discrete-wave periods *l*.

## The Model and Main Results

### Admissible configuration and configurational energy

We consider chains consisting of $n \in \mathbb {N}$ particles and corresponding deformations $y: \lbrace 1,\ldots , n \rbrace \rightarrow \mathbb {R}^2$. We write $y_i = y(i)$ for $i =1,\ldots ,n$ and introduce the set of *admissible configurations* by

1 $$\begin{aligned} \begin{aligned}&\mathcal {A}_n = \lbrace y :\lbrace 1,\ldots , n \rbrace \rightarrow \mathbb {R}^2 \, | \ |y_i - y_j| > 1.5 \ \text { for } \ i,j: \ |i - j| \ge 2, \\&|y_i - y_{i+1}| \le 1.5 \ \text { for } \ i=1,\ldots ,n-1 \rbrace . \end{aligned} \end{aligned}$$

The conditions above ensure that only consecutive points in the chain are *bonded*. In particular, apart from $i=1$ and $i=n$, each atom is bonded to exactly two other particles. Here, the value 1.5 is chosen for definiteness only.

For two vectors $a_1,a_2 \in \mathbb {R}^2$ we let $\sphericalangle (a_1,a_2)\in [0,2\pi )$ be the angle between $a_1$ and $a_2$, measured counterclockwisely. We define the *bond lengths* and *angles* of the chain by

2 $$\begin{aligned} b_i&= |y_i - y_{i+1}| \ \text { for } \ i=1,\ldots , n-1, \nonumber \\ \varphi _i&= \sphericalangle (y_{i-1} - y_i, y_{i+1} - y_i) \ \text { for } \ i=2,\ldots ,n-1. \end{aligned}$$

In the following we introduce the *configurational energy* $E_n$ *of a chain*, and we detail the hypotheses which we assume on $E_n$ throughout the paper. The energy is given by the sum of two contributions, respectively accounting for *two-body and three-body interactions among particles* that are respectively modulated by the potentials $v_2$ and $v_3$, see (3) and (7).

We assume that the *two-body potential* $v_2:(0,\infty )\rightarrow [-1,\infty )$ is smooth and attains its minimum value only at 1 with $v_2(1) = -1$ and $v''_2(1)>0$. Moreover, we suppose that $v_2$ is strictly increasing right of 1. Referring to the modeling of graphene sheets [11], this potential models the effective interaction between different graphene-lattice cells, favoring a specific distance of cell centers, here normalized to 1.

The *three-body potential* $v_3: [0,2\pi ]\rightarrow [0,\infty )$ is assumed to be smooth and symmetric around $\pi$, namely $v_3(\pi -\varphi )=v_3(\pi +\varphi )$. Moreover, we suppose that the minimum is attained only at $\pi$ with $v_3(\pi ) = v_3'(\pi ) = v''_3(\pi ) = v_3'''(\pi ) = 0$, and $v_3''''(\pi ) >0$. With reference to the modeling of graphene sheets, the latter potential describes the energy associated with the flatness of adjacent graphene-lattice cells [11]. In particular, $v_3$ is not related to angles between bonded carbon atoms but contributes an effective descriptor of flatness of cells. The reader is referred to [11, Section 5] and in particular to formula [11, (5.2)] for a discussion of this term.

We introduce a configurational energy by

3 $$\begin{aligned} E^{\mathrm{red}}_n(y) = \sum _{i=2}^{n-1}E_{\mathrm{cell}}(y_{i-1}, y_i,y_{i+1}) \end{aligned}$$

where the *cell energy* is defined as

4 $$\begin{aligned} E_{\mathrm{cell}}(y^1,y^2,y^3)&= v_2(|y^2-y^1|) + v_2(|y^3-y^2|) \nonumber \\&\quad + v_3(\sphericalangle (y^3-y^2,y^1-y^2) ) + \rho v_2(|y^3-y^1|) \end{aligned}$$

for $y^1,y^2,y^3 \in \mathbb {R}^2$. The constant $\rho > 0$ will be chosen to be suitably small later on. More precisely, one could reformulate the whole theory by prescribing a single two-body potential $\tilde{v}_2$ and letting

5 $$\begin{aligned} E_{\mathrm{cell}}(y^1,y^2,y^3)&= {\tilde{v}}_2(|y^2-y^1|) + \tilde{v}_2(|y^3-y^2|) + v_3(\sphericalangle (y^3-y^2,y^1-y^2) ) \nonumber \\&\quad + \tilde{v}_2(|y^3-y^1|). \end{aligned}$$

In this setting, the dimensionless constant $\rho >0$ would measure the ratio between the energetic contributions of first and second neighbors. (Specifically, ${\tilde{v}}_2(1)=-1$ and $|\tilde{v}_2(2)| \le \rho$ in a neighborhood of 2.) Since our analysis is largely based on the smallness of such ratio, we prefer to highlight this in the notation and stick to the equivalent form in (4).

Since in the sequel we will consider also a more general energy, the configurational energy (3) is called the *reduced energy*. Let us mention that due to the fact that $E^{\mathrm{red}}_n$ is written as a sum over cell energies, the two-body contributions at the left and right end of the chain are counted only once and not twice. However, since we focus on the case of large numbers of particles *n* and we are not interested in describing the fine geometry close to the ends of the chain, this effect will be negligible for our analysis.

Our first result addresses the configurations with minimal reduced energy. In particular, we check that all configurations minimizing the reduced energy have bonds of equal length and show exactly two possible bond angles.

#### Theorem 2.1

(Minimizers for the reduced energy). Let $\rho >0$ be small depending only on $v_2$ and $v_3$. Then there exist $e_{\mathrm{cell}} \in \mathbb {R}$, $0<{\bar{b}}<1$, and ${\bar{\psi }} \in (0,\pi /8)$ such that

$$\begin{aligned} \min _{y \in \mathcal {A}_n} E^{\mathrm{red}}_n(y) = (n-2)e_{\mathrm{cell}} \end{aligned}$$

and each configuration $y \in \mathcal {A}_n$ with minimal energy satisfies $b_i = {\bar{b}}$ for $i=1,\ldots ,n-1$ and $\varphi _i = \pi + {\bar{\psi }}$ or $\varphi _i = \pi - {\bar{\psi }}$ for $i=2,\ldots ,n-1$.

The result relies on the properties of the cell energy (4) and is proved in Sect. 3. We observe that there are many minimizers of the energy with very different geometries, see Fig. 3. In particular, to exclude certain geometries, in the following we will take given boundary conditions into account. This is realized by specifying the length of the chain in direction $e_1$. Indeed, let us fix the *straining parameter* $\mu$ in the set of admissible values *M*, with $M\subset (2/3,1)$ being a closed interval, and define

6 $$\begin{aligned} \mathcal {A}_n(\mu ) = \lbrace y \in \mathcal {A}_n| \ (y_n -y_1) \cdot e_1 = (n-1)\mu \rbrace . \end{aligned}$$

Note that the length $|y_n - y_1|$ of a minimizer of the reduced energy is necessarily strictly smaller than $n-1$, for ${{\bar{b}}}<1$ and ${{\bar{\psi }}} >0$. This implies that the choice of values of $\mu$ close to 1 in $\mathcal {A}_n(\mu )$ actually corresponds to *straining* the chain.

**Fig. 4:**
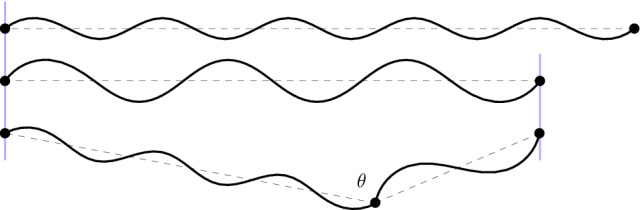
Almost minimizers of (3) consisting of single-period waves with different wavelengths (or in other words: different discrete-wave periods), represented by smooth waves for illustration purposes. Observe that the second and third configuration have different global geometries in spite of accommodating the same boundary conditions. The last configuration is only an almost minimizer since the angle $\theta$ is not $\pi \pm {\bar{\psi }}$

Even by restricting to the special subclass $\mathcal {A}_n(\mu )$, (almost) minimizers of (3) may have very different geometries, see Fig. 4.

To investigate the qualitative differences and different geometries of various configurations with (almost) minimal reduced energy in more detail, we now introduce a general, refined energy. For $y \in \mathcal {A}_n$ we let

7 $$\begin{aligned} E_n(y) = E^{\mathrm{red}}_n(y) + {\bar{\rho }} \sum _{i=1}^{n-3} v_2(|y_i -y_{i+3}|). \end{aligned}$$

The term on the right accounts for *longer-range interactions*. The constant ${\bar{\rho }}>0$ will be chosen suitably small with respect to $\rho$ later on, again reflecting the different relevance of the different contributions. We note that we could take more general interactions into account, but the contributions of third neighbors are already sufficient for our subsequent analysis and here we prefer simplicity rather than generality. Let us also mention that a reformulation of (7) in terms of a single two-body potential ${\tilde{v}}_2$, similar to (5), is possible.

### Characterization of minimal energy

We will now identify the minimal energy $E_n$ for given $\mu \in M$. We set

8 $$\begin{aligned} E_{\mathrm{min}}^{n,\mu } = \frac{1}{n-2} \min _{y \in \mathcal {A}_n(\mu )}E_n(y). \end{aligned}$$

#### Theorem 2.2

(Characterization of the minimal energy). For $\rho$ and ${\bar{\rho }}/\rho$ small enough (depending on $v_2$, $v_3$, and *M*) we find a constant $e^{\mathrm{gen}}_{\mathrm{cell}} \in \mathbb {R}$ and an increasing, convex, piecewise affine function $e_\mathrm{range}: M \rightarrow \mathbb {R}$, both only depending on $v_2,v_3$, $\rho$, and ${\bar{\rho }}$, such that

9 $$\begin{aligned} |e^{\mathrm{gen}}_{\mathrm{cell}} + {\bar{\rho }} e_{\mathrm{range}}(\mu ) - E_\mathrm{min}^{n,\mu } | \le c \big ( {\bar{\rho }}^2+ 1/n \big ) \end{aligned}$$

for all $\mu \in M$, where $c=c(v_2,v_3,\rho )>0$.

The energy has a *zero order term* $e^{\mathrm{gen}}_{\mathrm{cell}}$ which is constant for all values $\mu \in M$ and is a small perturbation of $e_{\mathrm{cell}}$ given in Theorem 2.1, i.e., $|e^{\mathrm{gen}}_{\mathrm{cell}} - e_{\mathrm{cell}}| \le c{\bar{\rho }}$. Differences in the minimal energy in terms of $\mu$ appear only in the *first order term* ${\bar{\rho }} e_\mathrm{range}$ which is associated to the longer-range interactions. For the exact definitions of $e^{\mathrm{gen}}_{\mathrm{cell}}$ and $e_\mathrm{range}$ we refer to (54) and (58) below, respectively. For an illustration of the graph of the function $e_{\mathrm{range}}$ we refer to Fig. 10.

In Theorem 2.3 below we will see that almost minimizers of the minimization problem (8) can be interpreted as *waves* (in a discrete sense). Then, ${\bar{\rho }} e_{\mathrm{range}}$ is essentially related to the *wavenumber* of the minimizer. In particular, smaller values of $\mu$ correspond to a smaller wavenumber or, respectively, to a larger *wavelength*. Compare also the first and the second configuration in Fig. 4. Roughly speaking, this effect corresponds to the waves having ‘constant curvature’, induced by the angle ${\bar{\psi }}$ from Theorem 2.1. In this context, the finite set

10 $$\begin{aligned} M_{\mathrm{res}} = \lbrace \mu \in M| \ e_{\mathrm{range}} \text { is not differentiable in } \mu \rbrace \end{aligned}$$

of *resonant lengths* plays a pivotal role since for $\mu \in M_{\mathrm{res}}$ minimizers of (8) are (almost) periodic waves, cf. Theorem 2.3 below.

We remark that the minimal energy can be characterized only up to small error terms of the form 1/*n* and ${\bar{\rho }}^2$. The term 1/*n* accounts for boundary effects at the left and right end of the chain, induced by the longer-range interactions. The term ${\bar{\rho }}^2$ on the right-hand side of (9) reflects the fact that periodic waves with different wavelengths lead to a different longer-range interaction. This effect will be discussed in more detail in Lemma 5.3.

### Characterization of almost minimizers

We now proceed with the characterization of almost minimizers. Recalling (2) we define

11 $$\begin{aligned} \mathcal {C}(y):= \lbrace i \in \lbrace 2,\ldots ,n-2 \rbrace \, | \ \varphi _i > \pi , \ \varphi _{i+1} < \pi \rbrace , \end{aligned}$$

which can be interpreted as particles where the chain ‘changes its curvature’. For convenience, we write

$$\begin{aligned} \mathcal {C}(y) = \lbrace i_1,\ldots , i_{N(y)} \rbrace \end{aligned}$$

for a strictly increasing sequence of integers, where $N(y) \in \mathbb {N}$ depends on *y*. We will interpret $|y_{i_{k+1}} - y_{i_k}|$, $k=1,\ldots ,N(y)-1$, as the wavelength of a wave.

In the following, we say $y \in \mathcal {A}_n(y)$ is an *almost minimizer* of (8) if

12 $$\begin{aligned} \frac{1}{n-2}E_n(y) \le E_{\mathrm{min}}^{n,\mu } + c \big ( {\bar{\rho }}^2+ 1/n\big ), \end{aligned}$$

where *c* is the constant from Theorem 2.2. We now present two results on the characterization of almost minimizers, starting from the resonant case $\mu \in M_{\mathrm{res}}$.

#### Theorem 2.3

(Characterization of almost minimizers, $\mu \in M_{\mathrm{res}}$). Let $M_{\mathrm{res}}$ be defined in (10) and let $\varepsilon >0$. Then for $\rho$ and ${\bar{\rho }}/\rho$ small enough, depending on $v_2$, $v_3$, and *M*, there are a finite, decreasing sequence $\lambda (\mu ), \mu \in M_{\mathrm{res}}$, only depending on $v_2$, $v_3$, $\rho$, and a constant $c=c(v_2,v_3,\rho ,\varepsilon )>0$ such that following holds for all $n \ge {\bar{\rho }}^{-2}$:

For each $\mu \in M_{\mathrm{res}}$ every almost minimizer $y \in \mathcal {A}_n(\mu )$ of (8), with $\mathcal {C}(y) = \lbrace i_1,\ldots , i_{N(y)}\rbrace$, satisfies

13 $$\begin{aligned} \big ||y_{i_{k+1}} - y_{i_k}| - \lambda (\mu )\big | \le \varepsilon \end{aligned}$$

for $$ i_k \in \mathcal {K} \subset \mathcal {C}(y)$$, where

14 $$\begin{aligned} \# (\mathcal {C}(y) \setminus \mathcal {K}) / n \le c{\bar{\rho }}. \end{aligned}$$

Theorem 2.3 states that, despite of nonuniqueness, the minimizers can be characterized in terms of the *wavelength* $\lambda (\mu )$. We remark that the parts of the chain satisfying (13) correspond to a fixed number of bonds, also referred to * discrete-wave period* in the following, i.e., $l_\mu :=i_{k+1} - i_k$ is constant for all $i_k \in \mathcal {K}$. More precisely, we will show below in Lemma 4.3 that the connection between $\mu$, the wavelength, and the discrete-wave period is given by the formula

15 $$\begin{aligned} \lambda (\mu )= \mu l_\mu = 2{\bar{b}}\sin ({\bar{\psi }}l_\mu /4)/\tan ({\bar{\psi }}/2) \end{aligned}$$

with the bond length ${\bar{b}}$ and the angle ${\bar{\psi }}$ from Theorem 2.1. Notice that the fact that the sequence $\lambda (\mu )$ is decreasing in $\mu$ (or equivalently, $l_\mu$ is decreasing in $\mu$) is in accordance with the above remark that smaller values of $\mu$ correspond to larger wavelengths, see again Fig. 4.

Let us remark that the assumption $n \ge {\bar{\rho }}^{-2}$ can be dropped at the expense of a more complicated estimate (14). We however prefer to keep this assumption for simplicity since we are indeed interested in the case of a large number of particles.

In Corollary 6.1, we will explicitly provide an example of a chain involving waves of different discrete-wave periods in order to show that in general it is energetically favorable that $\# (\mathcal {C}(y) \setminus \mathcal {K})$ is positive. In particular, minimizers are not expected to be periodic, but only periodic ‘outside of a small set’, controlled in terms of ${\bar{\rho }}$. In particular, Corollary 6.1 will show that (a) the minimal energy in Theorem 2.2 can be characterized only up to a higher order error term of the form ${\bar{\rho }}^2$ and that (b) the characterization given in Theorem 2.3, see (14), is sharp.

Let us now drop the resonance assumption and present a characterization result for almost minimizers for general $\mu$.

#### Theorem 2.4

(Characterization of almost minimizers, general case). Let $M \subset (2/3,1)$ be the closed interval introduced right before (6) and let $\varepsilon >0$. For $\rho$ and ${\bar{\rho }}/\rho$ small enough, let $\lambda (\mu ), \mu \in M_{\mathrm{res}}$, be the sequence and let $c=c(v_2,v_3,\rho ,\varepsilon )>0$ be the constant from Theorem *2.3*. Suppose that $n \ge {\bar{\rho }}^{-2}$.

Let $\mu \in M$ with $\mu \in [\mu ',\mu '']$ for $\mu ',\mu '' \in M_{\mathrm{res}}$ with $(\mu ',\mu '') \cap M_{\mathrm{res}} = \emptyset$. Then every almost minimizer $y\in \mathcal {A}_n(\mu )$ of (8), with $\mathcal {C}(y) = \lbrace i_1,\ldots , i_{N(y)}\rbrace$, satisfies

16 $$\begin{aligned} \begin{aligned}&\big ||y_{i_{k+1}} - y_{i_k}| - \lambda (\mu ')\big | \le \varepsilon \ \text { for } i_k \in \mathcal {K}', \\&\big ||y_{i_{k+1}} - y_{i_k}| - \lambda (\mu '')\big | \le \varepsilon \ \text { for } i_k \in \mathcal {K}'', \end{aligned} \end{aligned}$$

where $$\mathcal {K}', \mathcal {K}'' \subset \mathcal {C}(y)$$ satisfy

17 $$\begin{aligned} |\sigma \# \mathcal {C}(y) - \#\mathcal {K}'| / n \le c{\bar{\rho }}, \ \ \ \ |(1-\sigma ) \# \mathcal {C}(y) - \#\mathcal {K}''| / n \le c {\bar{\rho }}, \end{aligned}$$

where $\sigma$ only depends on $\mu$, but not on *y*. In particular, in accordance with Theorem *2.3*, we have $\sigma = 1$ for $\mu = \mu '$ and $\sigma = 0$ for $\mu = \mu ''$.

This result states that, for $\mu$ between two resonant lengths $\mu '$ and $\mu ''$, the almost minimizer shows essentially the two wavelengths $\lambda (\mu ')$ and $\lambda (\mu '')$ in proportion $\sigma$ and $1-\sigma$, respectively, where $\sigma$ depends just on $\mu$.

The proofs of Theorems 2.3–2.4 are contained in Sects. 4–6. We start with the analysis of a single-period problem in Sect. 4, move on to the problem of multiple periods in Sect. 5, and finally give the proof of the main results in Sect. 6. We warn the Reader that in the following all generic constants may depend on the potentials $v_2$ and $v_3$ without explicit mentioning. Dependencies on other constants such as $\rho$, ${\bar{\rho }}$, or $\varepsilon$, will always be indicated in brackets after the constant. Moreover, we will often use the notation $\lfloor x \rfloor = \max \{z \in \mathbb {Z}\, : \, z \le x\}$ and $\lceil x \rceil = \min \{z \in \mathbb {Z}\, : \, x \le z\}$ for $x\in \mathbb {R}$.

### An illustration on a simpler model

**Fig. 5:**
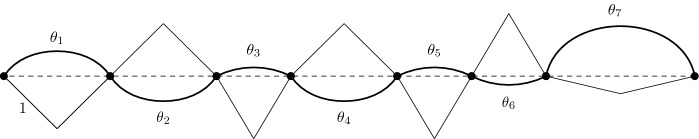
Configurations in the simplified setting of Sect. 2.4: juxtaposition of arcs of a circle of radius 1, determined by the respective lengths $\theta _1, \dots , \theta _7 \in [0,\pi )$

We close this section by discussing a simpler model, where configurations are made of the juxtaposition of arcs of a circle of a fixed radius, see Fig. 5.

This continuous, simplified setting is still capable of illustrating some of the main features of the general model. In particular, it allows to identify an optimal wavelength, independently of the sample size. On the other hand, it avoids many technicalities and, correspondingly, it is much less detailed.

As said, configurations correspond to curves consisting of a finite number of arcs of a circle, whose radius is normalized to 1, and having non-overlapping secants on some given axis. The configuration is hence identified by the lengths $\{\theta _1, \dots ,\theta _k\} \in [0,\pi )^k$ of the corresponding arcs. The total length of the curve is given by

$$\begin{aligned} \Theta = \sum _{i=1}^k \theta _i . \end{aligned}$$

On the other hand, the projection of the curve on the axis has length

$$\begin{aligned} \Pi =\sum _{i=1}^k 2 \sin (\theta _i/2). \end{aligned}$$

Note that, for all $k \in \mathbb {N}$ given, the maximal length of the projection $\Pi$ is achieved by the configuration made of *k* equal arcs with length $\Theta /k$. In fact, the concavity of $\sin$ on $[0,\pi ]$ entails that $\Pi \le 2k \sin (\Theta /(2k))$, where equality holds iff $\theta _i = \Theta /k$ for all *i*.

We now reformulate the variational problems by restricting to those curves of fixed length $\Theta >0$ fulfilling the boundary condition $\Pi = \mu \Theta$, where the given straining parameter $\mu \in (0,1)$ has the exact same meaning as in (6). As all arcs have the same curvature, to minimize the energy in this case corresponds to minimize the number of curvature changes, i.e., $k-1$. Let $f:[ 2/\pi ,1 ] \rightarrow [0,\pi /2]$ be the inverse function of $\tau \mapsto \sin (\tau )/\tau$, which is concave and strictly decreasing. The minimal value $k_{\mathrm{min}}$ can be computed in terms of $\mu$ as

$$\begin{aligned} k_{\mathrm{min}} = \left\lceil \frac{\Theta }{2f(\mu )} \right\rceil . \end{aligned}$$

In case $\mu$ is such that $\Theta /(2f(\mu )) \in \mathbb {N}$, we have that the configuration with minimal energy is the juxtaposition of $k_{\mathrm{min}}$ arcs of equal length $\theta ^*:= \Theta /k_\mathrm{min}$. For all $\mu$ which do not belong to such discrete set, the optimal curve consists of $k_{\mathrm{min}}$ arcs, which necessarily cannot be all of equal length.

Note that the optimal arc length $\theta ^*$ is invariant with respect to the length $\Theta$ of the curve: given $\mu$ with $\Theta /(2f(\mu )) \in \mathbb {N}$, among curves with length $\Theta ' := \Theta m/k_{\mathrm{min}}$ for $m \in \mathbb {N}$, the optimal configuration is the juxtaposition of *m* arcs of the same optimal length $\Theta '/m= \Theta / k_{\mathrm{min}} =\theta ^*$. This in particular illustrates in this simplified setting the onset of a specific, sample-size independent optimal wavelength.

## The Cell Problem

In this short section we focus on the cell energy (4) and prove Theorem 2.2. Let us firstly note that the cell energy can be written equivalently in terms of bond lengths and angles. More precisely, we introduce

$$\begin{aligned} \tilde{E}_{\mathrm{cell}}(b_1,b_2,\varphi ):= & {} E_{\mathrm{cell}}(y^1,y^2,y^3) = v_2(b_1) + v_2(b_2) \\&+ \rho v_2\Big (\sqrt{b_1^2 + b_2^2 - 2b_1b_2\cos \varphi } \Big ) + v_3(\varphi ), \end{aligned}$$

where $b_1 = |y^1-y^2|$, $b_2 = |y^2-y^3|$, and $\varphi = \sphericalangle (y^3-y^2,y^1-y^2)$. Owing to this notation, we can now state the following.

### Lemma 3.1

(Minimizers and convexity of the cell energy). We have that

(i) For $\rho >0$ small enough (depending only on $v_2$ and $v_3$) there exist $0< {\bar{b}} < 1$ and ${\bar{\psi }} \in (0,\pi /8)$ such that the minimizers of $\tilde{E}_{\mathrm{cell}}$ are given by
  $$\begin{aligned} ({\bar{b}}, {\bar{b}}, \pi + {\bar{\psi }}) \ \ \ \ \text {and} \ \ \ \ ({\bar{b}}, {\bar{b}}, \pi - {\bar{\psi }}). \end{aligned}$$
(ii) The cell energy $\tilde{E}_{\mathrm{cell}}$ is smooth in a neighborhood of the minimizers and there exists $c_{\mathrm{conv}}=c_\mathrm{conv}(\rho )>0$ such that its Hessian at the minimizers satisfies
  18 $$\begin{aligned} D^2 \tilde{E}_{\mathrm{cell}} ({\bar{b}},{\bar{b}},\pi \pm {\bar{\psi }}) \ge c_{\mathrm{conv}}{\varvec{I}}, \end{aligned}$$
  where ${\varvec{I}} \in \mathbb {R}^{3 \times 3}$ denotes the identity matrix.

### Proof

Ad *(i)*. Fix $\varepsilon >0$ small. Since for $\rho =0$ the energy is uniquely minimized by $(1,1, \pi )$, for $\rho$ small (depending on $\varepsilon$) the minimizers of $\tilde{E}_{\mathrm{cell}}$ lie in $(1-\varepsilon ,1+\varepsilon )^2 \times (\pi -\varepsilon ,\pi +\varepsilon )$. For all fixed $(b_1,b_2) \in (1-\varepsilon ,1+\varepsilon )^2$, we consider the mapping $f(\varphi ) = \tilde{E}_{\mathrm{cell}}(b_1,b_2, \varphi )$ for $\varphi \in (\pi -\varepsilon ,\pi + \varepsilon )$. The second derivative of *f* reads as

$$\begin{aligned} f''(\varphi )&= v_3''(\varphi ) + \rho v_2''\Big (\sqrt{b_1^2 + b_2^2 - 2b_1b_2\cos \varphi } \Big ) \ \frac{ (b_1b_2\sin \varphi )^2}{b_1^2 + b_2^2 - 2b_1b_2\cos \varphi } \\&\ \ \ + \rho v_2'\Big (\sqrt{b_1^2 + b_2^2 - 2b_1b_2\cos \varphi } \Big ) \ \frac{(b_1^2 + b_2^2 - 2b_1b_2\cos \varphi )b_1b_2\cos \varphi - (b_1b_2\sin \varphi )^2}{(b_1^2 + b_2^2 - 2b_1b_2\cos \varphi )^{3/2}}. \end{aligned}$$

Consequently, $f''(\pi ) < 0$ since $v_2$ is strictly increasing right of 1 and $v_3''(\pi )=0$. Moreover, as $v_3$ is symmetric around $\pi$, *f* is symmetric around $\pi$ as well. Thus, it suffices to identify a unique minimizer of $(b_1,b_2,\psi ) \mapsto \tilde{E}_{\mathrm{cell}}(b_1,b_2,\pi + \psi )$ on $(1-\varepsilon ,1+\varepsilon )^2 \times (0,\varepsilon )$. After a transformation, this is equivalent to show that

19 $$\begin{aligned} G(b_1,b_2,\theta ) = \tilde{E}_{\mathrm{cell}}(b_1,b_2, \pi + \sqrt{\theta }) \end{aligned}$$

has a unique minimizer on $D^\varepsilon := (1-\varepsilon ,1+\varepsilon )^2 \times (0,\varepsilon ^2)$.

We set $g_1(b_1,b_2,\theta ) = v_2(b_1) + v_2(b_2) + v_3(\pi + \sqrt{\theta })$ and $g_2 = \big (G - g_1\big )/\rho$. Let the functions $\lambda _1$ and $\lambda _2$ denote the smallest eigenvalues of $D^2 g_1$ and $D^2 g_2$, respectively. Using a Taylor expansion for $v_3$ around $\pi$, we compute $D^2g_1(1,1,0) = \mathrm{diag}(v_2''(1),v_2''(1),v_3''''(\pi )/12)$. Thus, for $\varepsilon$ small enough, $\lambda _1$ is positive on $D^\varepsilon$ by the assumptions on $v_2$ and $v_3$. Consequently, for $\rho$ small enough, depending only on $v_2$ and $v_3$, we find a constant $c_G>0$ such that

20 $$\begin{aligned} \lambda _1(b_1,b_2,\theta ) + \rho \lambda _2 (b_1,b_2,\theta )\ge c_G \end{aligned}$$

for all $(b_1,b_2,\theta ) \in D^\varepsilon$. For such small $\rho$, *G* is therefore strictly convex on $D^\varepsilon$.

This implies that the minimizer of *G* is uniquely determined and, by the symmetry of *G* in the variables $b_1$ and $b_2$, it has the form $({\bar{b}}, {\bar{b}}, {\bar{\theta }} )$. We conclude that $\tilde{E}_{\mathrm{cell}}$ is minimized exactly at $({\bar{b}},{\bar{b}}, \pi \pm {\bar{\psi }})$ with ${\bar{\psi }} = \sqrt{{\bar{\theta }}}$. The first order optimality condition $\partial _{b_1} G({\bar{b}},{\bar{b}},{\bar{\theta }}) = 0$ implies

$$\begin{aligned} v_2'({\bar{b}}) + \rho v_2'\big ({\bar{b}}\sqrt{2(1-\cos {\bar{\varphi }})}\big ) \ \sqrt{(1-\cos {\bar{\varphi }})/2} = 0, \end{aligned}$$

where ${\bar{\varphi }} = \pi + {\bar{\psi }}$. Since ${\bar{b}}\sqrt{2(1-\cos {\bar{\varphi }})}>1$ for $\varepsilon >0$ small, we get ${\bar{b}} < 1$ by the assumptions on $v_2$. Similarly, possibly taking $\varepsilon$ small enough, we find ${\bar{\psi }} \in (0,\pi /8)$.

Ad *(ii)*. The smoothness of the cell energy $\tilde{E}_\mathrm{cell}$ in a neighborhood of the minimizers follows directly from the assumptions on $v_2$ and $v_3$. For brevity we set ${\varvec{d}} = (b_1,b_2,\varphi )$ and $T({\varvec{d}}) = (b_1,b_2,(\varphi -\pi )^2)$. For $\varphi$ in a neighborhood of $\pi + {\bar{\psi }}$ we can write $\tilde{E}_{\mathrm{cell}}({\varvec{d}}) = G(T({\varvec{d}}))$ with *G* from (19). For each ${\varvec{v}} \in \mathbb {R}^3$, an elementary computation yields $D\tilde{E}_{\mathrm{cell}}({\varvec{d}}) {\varvec{v}} = DG (T({\varvec{d}})) DT({\varvec{d}}){\varvec{v}}$ and

$$\begin{aligned} D^2 \tilde{E}_{\mathrm{cell}}({\varvec{d}})[{\varvec{v}},{\varvec{v}}] = D^2G (T({\varvec{d}})) [D T({\varvec{d}}){\varvec{v}}, DT({\varvec{d}}){\varvec{v}}] + DG (T({\varvec{d}})) D^2T({\varvec{d}})[{\varvec{v}},{\varvec{v}}]. \end{aligned}$$

Set ${\varvec{d}}_0 = ({\bar{b}},{\bar{b}},\pi + {\bar{\psi }})$. Since $DG(T({\varvec{d}}_0))=0$ by the first order optimality conditions, we obtain

$$\begin{aligned} D^2 \tilde{E}_\mathrm{cell}({\varvec{d}}_0)[{\varvec{v}},{\varvec{v}}] = D^2G (T({\varvec{d}}_0)) [DT({\varvec{d}}_0){\varvec{v}}, DT({\varvec{d}}_0){\varvec{v}}]. \end{aligned}$$

This together with (20) and the fact that $D T({\varvec{d}}_0) = \mathrm{diag}(1,1,2(\varphi -\pi ))$ yields (18) and concludes the proof. $\quad \square$

### Remark 3.2

(Smallness of ${\bar{\psi }}$) The proof shows that ${\bar{\psi }} \rightarrow 0$ as $\rho \rightarrow 0$. In the following sections, we will frequently assume that ${\bar{\psi }}$ is small with respect to constants depending on $v_2$, $v_3$, and the closed interval *M* introduced before (6). This will amount to choosing $\rho$ sufficiently small.

We conclude this section with the proof of Theorem 2.1.

### Proof of Theorem 2.1

The statement follows immediately from Lemma 3.1 and (3) with the constant $e_\mathrm{cell} = \tilde{E}_{\mathrm{cell}}({\bar{b}}, {\bar{b}},\pi + {\bar{\psi }})$. $\quad \square$

## The Single-Period Problem

The goal of this section is to consider chains $y \in \mathcal {A}_n$, *n* fixed and small, so that we expect minimizers to be represented by a wave consisting of one single period. In this section, we will only consider the reduced energy introduced in (3). We will first investigate the geometry and the length of configurations with minimal energy. Here, it will turn out that the analysis is considerably different for even and odd numbers of bonds. Afterwards, we study small perturbations of energy minimizers and show that the length excess can be controlled by the energy excess.

### Geometry and length of energy minimizers

We investigate the geometry and the length of configurations $y \in \mathcal {A}_n$ with minimal energy, i.e., $E^{\mathrm{red}}_n(y) = (n-2) e_{\mathrm{cell}}$, see Theorem 2.1. Let $n= l+1$, where *l* will stand for the * discrete-wave period*. Recall the definition of the bond lengths $b_i$ and the angles $\varphi _i$ in (2). Moreover, let ${\bar{b}}$ and ${\bar{\psi }}$ be the values found in Lemma 3.1. By $\mathcal {U}^l$ we denote the family of configurations $y \in \mathcal {A}_{l+1}$ such that the bond lengths coincide with that of minimizers of the cell energy, namely

21 $$\begin{aligned} b_i = {\bar{b}}, \ \ \ \ i=1,\ldots ,l, \end{aligned}$$

and such that there exists $i_0 \in \lbrace 2,\ldots ,l-1\rbrace$ with

22 $$\begin{aligned} \varphi _i = \pi - {\bar{\psi }} \ \ \ \text {for} \ \ \ i \in \lbrace 2,\ldots ,i_0 \rbrace , \ \ \ \ \ \varphi _i = \pi + {\bar{\psi }} \ \ \ \text {for} \ \ \ i \in \lbrace i_0+1,\ldots , l \rbrace . \end{aligned}$$

Note that, in particular, all configurations in $\mathcal {U}^l$ are minimizers of $E^{\mathrm{red}}_{l+1}$. Moreover, given the index $i_0$, the position of the points $y \in \mathcal {U}^l$ is determined uniquely up to a rotation and a translation. In particular, the *length* of the chain, denoted by $|y_{l+1} - y_1|$, is completely determined by the choice of $i_0$.

**Fig. 6:**
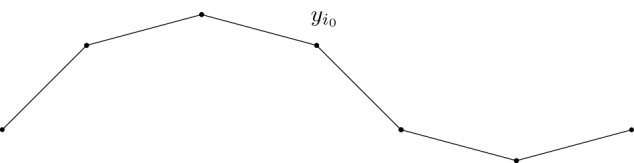
A single-period chain $y \in \mathcal {U}^6$

To identify the length of the chain, we will frequently use the formulas

23 $$\begin{aligned} \begin{aligned} \sum _{k=1}^m \sin (\theta - k{\bar{\psi }}) = \frac{\sin (m{\bar{\psi }}/2)}{\sin ({\bar{\psi }}/2)} \sin (\theta - (m+1){\bar{\psi }}/2),\\ \sum _{k=1}^m \cos (\theta - k{\bar{\psi }}) = \frac{\sin (m{\bar{\psi }}/2)}{\sin ({\bar{\psi }}/2)} \cos (\theta - (m+1){\bar{\psi }}/2) \end{aligned} \end{aligned}$$

for $\theta \in [0,2\pi )$ which can be derived by using a geometric series argument and the representations $\cos (x) = (e^{ix} + e^{-ix})/2$, $\sin (x) = (e^{ix} - e^{-ix})/2i$.

We recall that the angle between two vectors $a_1,a_2 \in \mathbb {R}^2$, measured counterclockwisely, is denoted by $\sphericalangle (a_1,a_2)$. We define the maximal possible discrete-wave period by

24 $$\begin{aligned} l_{\mathrm{max}} = 2\lceil 2\pi / {\bar{\psi }}\rceil - 4 \end{aligned}$$

and show that configurations $\mathcal {U}^l$ for $l \ge l_{\mathrm{max}}$ are not admissible.

#### Lemma 4.1

(Maximal discrete-wave period). The index $i_0$ from (22) satisfies $i_0 \le \lceil 2\pi /{\bar{\psi }}\rceil -2$ and $l+1 - i_0 \le \lceil 2\pi /{\bar{\psi }}\rceil -2$. In particular, we have $\mathcal {U}^l \cap \mathcal {A}_{l+1} = \emptyset$ for each $l \ge l_{\mathrm{max}}$.

#### Proof

Consider $y \in \mathcal {U}^l$. We first show that $y \notin \mathcal {A}_{l+1}$ if $i_0 \ge \lceil 2\pi /{\bar{\psi }}\rceil -1$. Let $j = \lceil 2\pi /{\bar{\psi }} \rceil$ and $\theta = \sphericalangle (e_1,y_2-y_1)$. We observe that $j-1 \le i_0$. Then we compute by (21), (22), and (23)

25 $$\begin{aligned} |y_j - y_1|&= {\bar{b}}\Big |\sum ^{j-1}_{i=1} \big (\cos (\theta + {\bar{\psi }} - i{\bar{\psi }}),\sin (\theta + {\bar{\psi }} -i{\bar{\psi }})\big )\Big | \nonumber \\&= {\bar{b}}\sin \big ((j-1){\bar{\psi }}/2 \big ) / \sin \big ({\bar{\psi }}/2\big )\le 1, \end{aligned}$$

where the last step follows from ${\bar{b}} \le 1$ (see Theorem 2.1) and $(j-1){\bar{\psi }}/2 \in [\pi -{\bar{\psi }}/2,\pi ]$. Thus, the assumption in (1) is violated and therefore $y \notin \mathcal {A}_{l+1}$. Likewise, we argue to find $y \notin \mathcal {A}_{l+1}$ if $l+1 - i_0 \ge \lceil 2\pi /{\bar{\psi }}\rceil -1$.

Combining the two conditions on the choice of $i_0$, we find that $l+1 = l+1-i_0 + i_0 \le 2\lceil 2\pi /{\bar{\psi }}\rceil -4$ for each $y \in \mathcal {U}^l \cap \mathcal {A}_{l+1}$. This implies $\mathcal {U}^l \cap \mathcal {A}_{l+1} = \emptyset$ for each $l \ge l_{\mathrm{max}}$. $\quad \square$

Recall that the length of the chain $|y_{l+1} - y_1|$ is completely determined by the choice of $i_0$ from (22). Thus, we can interpret $|y_{l+1} - y_1|$ as a function of $i_0$. More precisely, recalling also Lemma 4.1 we introduce

26 $$\begin{aligned}&\lambda ^l : \big \{ l+3 - \lceil 2\pi /{\bar{\psi }}\rceil , \ldots , \lceil 2\pi /{\bar{\psi }}\rceil - 2 \big \} \nonumber \\&\quad \cap \lbrace 2,\ldots ,l-1\rbrace \rightarrow (0,\infty ), \ \ \ \lambda ^l(i_0) = |y_{l+1} - y_1|, \end{aligned}$$

where $y \in \mathcal {U}^l \subset \mathcal {A}_{l+1}$ is a configuration satisfying (22) for $i_0$. The maximum of the function will be denoted by $\lambda ^l_\mathrm{max}$. Since the length is invariant under inversion of the order of the labels of the particles, we get $\lambda ^l(i) = \lambda ^l(l-i+1)$ for $i \le \lceil l/2 \rceil$.

After a rotation we may suppose that $({y}_{l+1} - {y}_1) \cdot e_2=0$ and $({y}_{l+1} - {y}_1) \cdot e_1>0$. In this case, letting

27 $$\begin{aligned} \phi _i = \sphericalangle (e_1, {y}_{i+1}- {y}_i) \end{aligned}$$

for $i=1,\ldots ,l$, we note that

28 $$\begin{aligned} |{y}_{l+1} - {y}_1| = \sum \nolimits _{i=1}^{l} {\bar{b}}\cos (\phi _i), \ \ \ \ \ \ \sum \nolimits _{i=1}^{l} \sin (\phi _i) = 0. \end{aligned}$$

We now determine the maximizer of $\lambda ^l$.

#### Lemma 4.2

(Maximizer of $\lambda ^l$). For $l \in \lbrace 2 ,\ldots , l_{\mathrm{max}} \rbrace$ the maximum of $\lambda ^l$ is attained exactly for $i_0 = \lceil l /2 \rceil$ and $i_0 = \lceil (l+1) /2 \rceil$.

#### Proof

We argue by contradiction. Suppose that the maximum is attained by a configuration ${y} \in \mathcal {U}^l$ with $i_0 \ne \lceil l /2 \rceil , \lceil (l+1) /2 \rceil$. After a rotation we may assume that $({y}_{l+1} - {y}_1) \cdot e_2=0$ and observe that (28) holds. In view of (22), a short computation yields

$$\begin{aligned} {\phi }_l = \big ({\phi }_1 + (l+1-2i_0) {\bar{\psi }}\big ) \mathrm{mod}2\pi \end{aligned}$$

with the angles $\phi _i$ defined in (27). Recall the symmetry $\lambda ^l(i) = \lambda ^l(l-i+1)$ for $i \le \lceil l/2 \rceil$, see right after (26). Using $i_0 \ne \lceil l /2 \rceil$, $i_0 \ne \lceil (l+1) /2 \rceil$, $i_0 \in [2,l-1] \cap [l+3 - \lceil 2\pi /{\bar{\psi }}\rceil , \lceil 2\pi /{\bar{\psi }}\rceil - 2]$, and distinguishing the cases whether *l* is larger than $\lceil 2\pi / {\bar{\psi }}\rceil$ or not, one may prove that $|({\phi }_1 - {\phi }_l)\mathrm{mod}2\pi | \ge 2{\bar{\psi }}$ after some tedious but elementary computations. This then implies $\cos ({\phi }_1) < \cos ({\phi }_l + {\bar{\psi }})$ or $\cos ({\phi }_l) < \cos ({\phi }_1 + {\bar{\psi }})$. After possibly inverting the labeling of the particles, it is not restrictive to assume that

29 $$\begin{aligned} \cos ({\phi }_1) < \cos ({\phi }_l + {\bar{\psi }}). \end{aligned}$$

We define a configuration ${\bar{y}} \in \mathcal {U}^l$ with index $\overline{i_0} = i_0 -1$ (see (22)) and ${\bar{\phi }}_1 = {\phi }_2$, where we indicate the angles in (27) corresponding to ${\bar{y}}$ by ${\bar{\phi }}_i$. Note that the configuration is characterized uniquely up to a translation. More precisely, we obtain

$$\begin{aligned} {\bar{\phi }}_i = {\phi }_{i+1} \ \ \ \text {for } i=1,\ldots ,l-1, \ \ \ \ \ {\bar{\phi }}_{l} = {\phi }_{l} + {\bar{\psi }}. \end{aligned}$$

By (28) and (29) this gives

$$\begin{aligned} |{\bar{y}}_{l+1}-{\bar{y}}_1| \ge \sum _{i=1}^l {\bar{b}}\cos ({\bar{\phi }}_i) = \sum _{i=1}^l {\bar{b}}\cos ({\phi }_i) + {\bar{b}}\cos ({\phi }_{l} + {\bar{\psi }}) - {\bar{b}}\cos ({\phi }_1)> | {y}_{l+1} - {y}_1|. \end{aligned}$$

Consequently, the length $|{y}_{l+1}-{y}_1|$ is not maximal among all configurations in $\mathcal {U}^l$. This contradicts the assumption and shows that the maximum is attained for $i_0 = \lceil l /2 \rceil$ or $i_0 = \lceil (l+1) /2 \rceil$. The fact that $\lambda ^l( \lceil l /2 \rceil ) = \lambda ^l(\lceil (l+1) /2 \rceil )$ by symmetry of $\lambda ^l$ concludes the proof. $\quad \square$

The previous result shows that for even $l \in 2\mathbb {N}\cap [2, l_{\mathrm{max}}]$ the maximum of $\lambda ^l$ is attained at $i_0 = l/2$, $i_0 = l/2+1$ and we call $\lambda ^l_{\mathrm{max}}= \lambda ^l(l/2)$ the *wavelength* of a wave with discrete-wave period *l*. The following lemma provides the relation between wavelength and even discrete-wave periods. Odd discrete-wave periods have to be treated differently, cf. Lemma 4.8 below.

#### Lemma 4.3

(Length for even discrete-wave periods). For all $l \in 2\mathbb {N}\cap [2, l_{\mathrm{max}}]$ we have $\lambda ^l_{\mathrm{max}} = 2{\bar{b}}\sin ({\bar{\psi }}l/4)/ \tan ({\bar{\psi }}/2).$

#### Proof

Fix $l \in 2\mathbb {N}\cap [2, l_{\mathrm{max}}]$ and consider a configuration $y \in \mathcal {U}^l$ as in (27) and (28) with $i_0 = l/2$. This leads to the choice $\phi _i = (l/4-i){\bar{\psi }}$ for $i \le l/2$ and $\phi _i = (-3l/4+i){\bar{\psi }}$ for $l/2+1 \le i \le l$. Indeed, we obtain $\sum _{i=1}^l \sin (\phi _i) = 0$ since $\phi _j = - \phi _{l/2+j}$ for $1 \le j \le l/2$. Moreover, we compute

$$\begin{aligned} \lambda ^l_{\mathrm{max}}&= \lambda ^l(l/2) = \sum _{i=1}^{l/2} {\bar{b}}\cos \big ((l/4-i){\bar{\psi }} \big ) \\&\quad + \sum _{i= l/2+1}^l {\bar{b}}\cos \big ((-3l/4+i){\bar{\psi }} \big ) = 2\sum _{i=1}^{l/2} {\bar{b}}\cos \big ((i-l/4){\bar{\psi }} \big ). \end{aligned}$$

With the help of (23), we then indeed get $\lambda ^l_{\mathrm{max}} = 2{\bar{b}}\sin ({\bar{\psi }}l/4)/ \tan ({\bar{\psi }}/2)$. $\quad \square$

#### Remark 4.4

The proof shows that a configuration $y \in \mathcal {U}^l$ as in (27) and (28) which realizes the maximal length $\lambda ^l_{\mathrm{max}}$ necessarily satisfies $\phi _1,\phi _l \in \lbrace (l/4-1){\bar{\psi }}, l{\bar{\psi }}/4 \rbrace$.

**Fig. 7:**
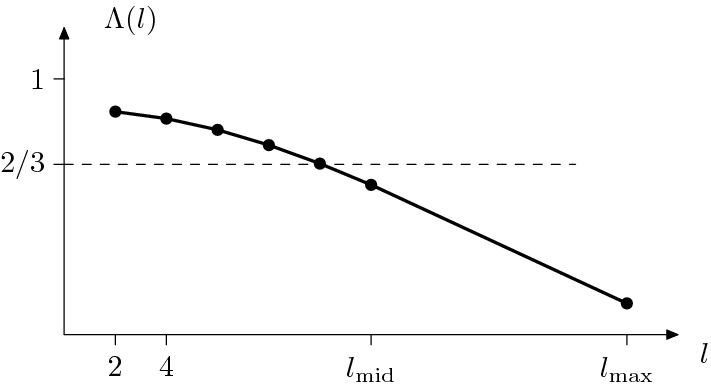
The normalized wavelength $\Lambda$

Let $l_{\mathrm{mid}} = \lfloor 6/{\bar{\psi }} \rfloor$ for brevity. In the following a distinguished role will be played by the *normalized wavelength* (normalized with respect to the number of bonds) $\Lambda : [2,l_{\mathrm{max}}]\rightarrow \mathbb {R}$, being the function which satisfies

30 $$\begin{aligned} \Lambda (l) = \frac{1}{l} \lambda ^l(l/2) = \frac{1}{l} \lambda _\mathrm{max}^l \end{aligned}$$

for $l \in 2 \mathbb {N}\cap [2,l_{\mathrm{mid}}]$, is affine on $[l-2,l]$, $l \in 2\mathbb {N}\cap [4, l_{\mathrm{mid}}]$, and affine on $[l_\mathrm{mid}-2,l_{\mathrm{max}}]$, see Fig. 7. The fact that the function is affine on the intervals between two even discrete-wave periods will be crucial (a) to identify the function $e_\mathrm{range}$ in Theorem 2.2 and (b) to give the characterization (17) in Theorem 2.4. Indeed, it will turn out that

$$\begin{aligned} \lbrace \mu = \Lambda (l)| \ l \in 2\mathbb {N}\cap [2, l_{\mathrm{mid}}] \rbrace \end{aligned}$$

is the set of *resonant lengths* $M_{\mathrm{res}}$ introduced in (10). We now study the properties of the normalized wavelength $\Lambda$.

#### Lemma 4.5

(Properties of the normalized wavelength $\Lambda$). The mapping $\Lambda$ is strictly decreasing and concave on $[2, l_{\mathrm{max}}]$. Moreover, $\Lambda (l) = \lambda ^l_{\mathrm{max}}/l$ for all $l \in 2\mathbb {N}\cap [2,l_{\mathrm{mid}}]$ and $\Lambda (l) \ge \lambda ^l_{\mathrm{max}}/l$ for all $l \in 2\mathbb {N}\cap (l_{\mathrm{mid}}, l_{\mathrm{max}}]$. Finally, for $\rho$ small enough we find $\Lambda ([2,l_{\mathrm{mid}}]) \supset (2/3,{\bar{b}}\cos ({\bar{\psi }}/2))$.

#### Proof

It is elementary to check that the mapping $f(x):=\sin (x)/x$ is strictly decreasing and concave on [0, 3/2]. Thus, recalling Lemma 4.3, the definition of $\Lambda$ in (30), and the fact that $l_{\mathrm{mid}}{\bar{\psi }}/4 \le 3/2$, we obtain that $\Lambda$ is strictly decreasing and concave. Moreover, one can check that

$$\begin{aligned} f(3/2) + f'(3/2)(x - 3/2) \ge f(x) \ \ \ \ \text {for all}{ x \in [3/2, \pi ].} \end{aligned}$$

From this we deduce that $\Lambda (l) \ge \lambda ^l_{\mathrm{max}}/l$ for all $l \in 2\mathbb {N}\cap (l_{\mathrm{mid}}, l_{\mathrm{max}}]$. Moreover, note that $\Lambda (l) = \lambda ^l_{\mathrm{max}}/l$ for all $l \in 2\mathbb {N}\cap [2,l_{\mathrm{mid}}]$ by definition. Finally, by Lemma 4.3 we compute $\Lambda (2) = {\bar{b}}\cos ({\bar{\psi }}/2)$ and $\Lambda (\lfloor 6/{\bar{\psi }} \rfloor ) = 2/3 \sin (3/2) {\bar{b}} + \mathrm{O}({\bar{\psi }})$, which shows that $\Lambda ([2,l_{\mathrm{mid}}]) \supset (2/3,{\bar{b}}\cos ({\bar{\psi }}/2))$ for $\rho$ (and thus ${\bar{\psi }}$, cf. Remark 3.2) sufficiently small. $\quad \square$

#### Remark 4.6

Clearly, as piecewise affine function, the normalized wavelength $\Lambda$ is not strictly concave. However, the strict concavity of $x \mapsto \sin (x)/x$ implies $\Lambda (\nu l_1 + (1-\nu )l_2) > \nu \Lambda (l_1) + (1-\nu )\Lambda (l_2)$ for all $\nu \in (0,1)$, whenever $\Lambda$ is *not* affine on $[l_1,l_2]$ with $l_1,l_2 \in [2,l_\mathrm{mid}]$. When we speak of *strict concavity of* $\Lambda$ in the following, we refer exactly to this property.

Before we proceed with the case of odd discrete-wave periods, we briefly note that configurations $\mathcal {U}^l$ can be connected to longer chains.

#### Remark 4.7

(Connecting two waves of maximal length) For $l \in 2\mathbb {N}\cap [2,l_{\mathrm{mid}}]$ choose the configuration $y^{\mathrm{max}, l} \in \mathcal {U}^l$ satisfying

31 $$\begin{aligned} y^{\mathrm{max},l}_1 = 0, \ \ \ \ \ \ y^{\mathrm{max}, l}_{l+1} = \lambda ^l_{\mathrm{max}} e_1 = l\Lambda (l)e_1 \end{aligned}$$

as well as $\sphericalangle (e_1, y^{\mathrm{max},l}_2 -y^{\mathrm{max},l}_1 ) = l {\bar{\psi }}/4$ and $\sphericalangle (e_1, y^{\mathrm{max},l}_{l+1} -y^{\mathrm{max},l}_l ) = (l/4-1) {\bar{\psi }}/4$ (see Remark 4.4). Consider the configuration $y: \lbrace 1, \ldots , 2l+1\rbrace \rightarrow \mathbb {R}^2$ defined by $y_i = y^{\mathrm{max}, l}_i$ for $i \in \lbrace 1,\ldots , l+1 \rbrace$ and $y_i = y^{\mathrm{max}, l}_{i -l} + l\Lambda (l)e_1$ for $i \in \lbrace l+2,2l+1 \rbrace$. Then recalling (21)–(22), we find that all bonds and angles of *y* (see (2)) satisfy $b_i = {\bar{b}}$ and ${{{\bar{\varphi }}}}_i = \pi \pm {\bar{\psi }}$. Thus, $E^\mathrm{red}_{2l+1}(y) = (2l-1)e_{\mathrm{cell}}$ with $e_{\mathrm{cell}}$ from Theorem 2.1.

**Fig. 8:**
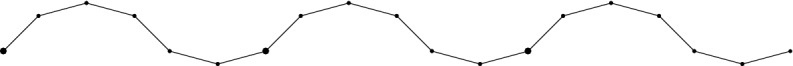
Connection of three waves $y^{\mathrm{max},l}$ minimizing the reduced energy

We now investigate in more detail the case of odd discrete-wave periods $l \in 2\mathbb {N}+1$. From Lemma 4.2 we get that the maximum of $\lambda ^l$ is attained exactly for $i_0 = (l+1) /2$. Without going into details, we remark that one can calculate for ${\bar{\psi }}$ sufficiently small that

$$\begin{aligned} \lambda ^l((l+1)/2) > \frac{1}{2} \Big ( (l-1) \Lambda (l-1) + (l+1)\Lambda (l+1) \Big ) + \frac{1}{2}(\Lambda (l-1)-\Lambda (l+1)) = l\Lambda (l). \end{aligned}$$

This in particular shows that the normalized wavelength $\Lambda$ does not capture correctly the wavelength for odd *l*. We hence proceed here by remarking that, under suitable conditions, the length for two consecutive waves with odd atomic period can be controlled in terms of the lengths of waves with even atomic period. This will eventually allow us to control the wavelength in terms of the normalized wavelength $\Lambda$ also for odd *l*.

More precisely, for odd $l_1,l_2 \in (2\mathbb {N}+1) \cap [2, l_\mathrm{max}]$ we let $y: \lbrace 1,\ldots , l_1+l_2 + 1 \rbrace \rightarrow \mathbb {R}^2$ be a configuration with $(y_1, \ldots ,y_{l_1+1}) \in \mathcal {U}^{l_1}$, $(y_{l_1+1} \ldots ,y_{l_1+l_2+1}) \in \mathcal {U}^{l_2}$, and the junction angle $\varphi _{l_1+1} - \pi = {\bar{\psi }}$ (see (2)). In view of (22), we find $(y_1, \ldots ,y_{l_1+2}) \in \mathcal {U}^{l_1+1}$, $(y_{l_1+2} \ldots ,y_{l_1+l_2+1}) \in \mathcal {U}^{l_2-1}$. Consequently, by the definition of the function $\lambda ^l$ in (26) and the triangle inequality we obtain

32 $$\begin{aligned} |y_{l_1+l_2+1} - y_1| \le \lambda _{\mathrm{max}}^{l_1+ 1} + \lambda _\mathrm{max}^{l_2-1}. \end{aligned}$$

This estimate can be obtained also for more general junction angles as the following lemma shows.

#### Lemma 4.8

(Length for odd discrete-wave periods). Let $l_1,l_2 \in (2\mathbb {N}+1) \cap [2, l_{\mathrm{max}}]$ and let $y: \lbrace 1,\ldots , l_1+l_2 + 1 \rbrace \rightarrow \mathbb {R}^2$ be a configuration with $(y_1, \ldots ,y_{l_1+2}) \in \mathcal {U}^{l_1+1}$, $(y_{l_1+2} \ldots ,y_{l_1+l_2+1}) \in \mathcal {U}^{l_2-1}$ and the junction angle $\varphi _{l_1+2} - \pi \in (1+ 2\mathbb {Z}){\bar{\psi }}$. Then

33 $$\begin{aligned} |y_{l_1+l_2+1} - y_1| \le \max _{t \in \lbrace -1,1\rbrace } \big (\lambda ^{l_1+t}_{\mathrm{max}} + \lambda ^{l_2-t}_{\mathrm{max}} \big ) -c_{\mathrm{mix}} {\varvec{1}}_{[0, \infty ) } (l_1-l_2), \end{aligned}$$

where $0< c_{\mathrm{mix}} < 1$ depends only on $l_{\mathrm{max}}$ (and thus only on $\rho$) and ${\varvec{1}}_A$ denotes the indicator function of a set *A*.

Note that the right-hand side of (33) is well defined in the sense that $l_1 + t, l_2 - t \le l_{\mathrm{max}}$ for $t \in \lbrace -1, 1\rbrace$ since $l_1,l_2 \le l_{\mathrm{max}}$ and $l_{\mathrm{max}}$ is even (see (24)). Notice that in contrast with the discussion before (32), the chains are connected at point $y_{l_1+2}$.

#### Proof

Let *y* be given as in the assumption. After a rotation we may suppose that $({y}_{l_1+l_2+1} - {y}_1) \cdot e_2=0$. Similarly to (27), we define the angles $\phi _i$, where the sum now runs from 1 to $l_1+l_2$. As $\varphi _{l_1+2} - \pi \in (1+ 2\mathbb {Z}){\bar{\psi }}$, we get

34 $$\begin{aligned} \phi _{l_1+1} - \phi _{l_1+2} \in (1+ 2\mathbb {Z}){\bar{\psi }}. \end{aligned}$$

As $(y_1, \ldots ,y_{l_1+2}) \in \mathcal {U}^{l_1+1}$ and $(y_{l_1+2} \ldots ,y_{l_1+l_2+1}) \in \mathcal {U}^{l_2-1}$, we derive similarly to (32)

$$\begin{aligned} |y_{l_1+l_2+1} - y_1| \le \lambda _{\mathrm{max}}^{l_1+1} + \lambda _{\mathrm{max}}^{l_2-1}. \end{aligned}$$

This shows (33) for $l_2>l_1$. From now on we suppose $l_1 \ge l_2$. In order to conclude the proof, it suffices to show the strict inequality

35 $$\begin{aligned} |y_{l_1+l_2+1} - y_1| < \max _{t \in \lbrace -1,1\rbrace } \big ( \lambda ^{l_1+t}_{\mathrm{max}} + \lambda ^{l_2-t}_{\mathrm{max}} \big ). \end{aligned}$$

Indeed, since the number of different admissible configurations (up to rigid motions) and the number of different $l_1,l_2$ is bounded by a number only depending on $l_{\mathrm{max}}$, we obtain the statement for a positive constant $c_{\mathrm{mix}}$, which only depends on $l_{\mathrm{max}}$ (and thus only on $\rho$).

It remains to show (35). First, suppose that $l_1-l_2 \ge 2$. Then we use Lemma 4.3, (24), and the strict concavity of $\sin$ on $[0,\pi ]$ to get

$$\begin{aligned} |y_{l_1+l_2+1} - y_1| \le \lambda _{\mathrm{max}}^{l_1+1} + \lambda _{\mathrm{max}}^{l_2-1}< \lambda _{\mathrm{max}}^{l_1-1} + \lambda _{\mathrm{max}}^{l_2+1}. \end{aligned}$$

If now $l_1 = l_2$, we assume by contradiction that the inequality in (35) was not strict. Equality would imply $({y}_{l_1+2} - {y}_1) \cdot e_2 = ({y}_{l_1+l_2+1} - {y}_{l_1+2}) \cdot e_2 = 0$, i.e., the two parts of the chain, lying in $\mathcal {U}^{l_1+1}$ and $\mathcal {U}^{l_2-1}$, respectively, satisfy (27) and (28). But then Remark 4.4 gives $\phi _{l_1+1} \in \lbrace (l_1/4-3/4){\bar{\psi }}, (l_1/4 + 1/4){\bar{\psi }} \rbrace$, $\phi _{l_1+2} \in \lbrace (l_2/4-5/4){\bar{\psi }}, (l_2/4 - 1/4){\bar{\psi }} \rbrace$. Since $l_1 = l_2$, we obtain a contradiction to (34). This establishes (35) and concludes the proof. $\quad \square$

### Small perturbations of energy minimizers

In this section, we investigate the length of single periods for configurations being small perturbations of energy minimizers. To this end, we introduce the set of small-perturbed chains

36 $$\begin{aligned} \mathcal {U}^l_{\delta } = \big \{ y \in \mathcal {A}_{l+1}| \ \exists \ {\bar{y}} \in \mathcal {U}^l: \ |b_i - {\bar{b}}| \le \delta , \ |\varphi _i - {\bar{\varphi }}_i| \le \delta \ \ \text {for all} \ i=1,\ldots ,l\big \}, \end{aligned}$$

where, as before, the angles $\varphi _i$ and ${\bar{\varphi }}_i$ corresponding to *y* and ${\bar{y}}$, respectively, are defined in (2). Likewise, the bond lengths will again be denoted by $b_i$. (For the angles the sum runs only from 2 to *l*.) In the following, we use the notation $(a)_+^2 = (\max \lbrace a,0\rbrace )^2$ for $a \in \mathbb {R}$ and the quantity $e_{\mathrm{cell}}$ from Theorem 2.1. Recall also $l_{\mathrm{max}}$ defined in (24). We first treat the case of even discrete-wave periods.

#### Lemma 4.9

(Energy excess controls length excess). There exist $\delta _0= \delta _0(\rho )>0$ and $C=C(\rho )>0$ such that for all $0 < \delta \le \delta _0$, for all $l \in 2\mathbb {N}\cap [2,l_{\mathrm{max}}]$, and all $y \in \mathcal {U}^l_\delta$ one has

$$\begin{aligned} E^{\mathrm{red}}_{l+1}(y) - (l-1)e_{\mathrm{cell}}\ge C \big (|y_{l+1} - y_1| - |{\bar{y}}_{l+1} - {\bar{y}}_1|\big )^2_+ \ge C \big (|y_{l+1} - y_1| - l\Lambda (l)\big )^2_+, \end{aligned}$$

where ${\bar{y}} \in \mathcal {U}^l$ is a configuration corresponding to *y* as given in the definition of $\mathcal {U}^l_\delta$.

#### Proof

Let $y \in \mathcal {U}^l_\delta$ and ${\bar{y}} \in \mathcal {U}^l$ be given. By Lemma 3.1 and a Taylor expansion we get for some $c>0$

37 $$\begin{aligned} E_{\mathrm{cell}}(y_{i-1},y_i,y_{i+1})&= \tilde{E}_{\mathrm{cell}}(b_{i-1},b_{i},\varphi _i) \ge e_{\mathrm{cell}} + \frac{c_{\mathrm{conv}}}{2} \nonumber \\&\quad \big (|b_{i-1} -{\bar{b}}|^2 + |b_{i} -{\bar{b}}|^2 + |\varphi _i - {\bar{\varphi }}_i|^2 \big )\nonumber \\&\quad - c\big (|b_{i-1} -{\bar{b}}|^3 + |b_{i} -{\bar{b}}|^3 + |\varphi _i - {\bar{\varphi }}_i|^3 \big )\nonumber \\&\ge e_{\mathrm{cell}} + \frac{c_{\mathrm{conv}}}{ 4 } \big (|b_{i-1} -{\bar{b}}|^2 + |b_{i} -{\bar{b}}|^2 + |\varphi _i - {\bar{\varphi }}_i|^2 \big ) \end{aligned}$$

for all $i=2,\ldots ,l$, where the last step follows with the definition of $\mathcal {U}^l_\delta$ and the choice $c\delta _0 \le c_{\mathrm{conv}}/ 4$. By (3) and Jensen’s inequality we get

38 $$\begin{aligned} E^{\mathrm{red}}_{l+1}(y)&= \sum _{i=2}^{l} E_{\mathrm{cell}}(y_{i-1},y_i,y_{i+1}) \ge (l-1)e_{\mathrm{cell}} + \frac{c_{\mathrm{conv}}}{ 4 } \Big ( \sum _{i=1}^{l}|b_{i} -{\bar{b}}|^2 + \sum _{i=2}^{l}|\varphi _i - {\bar{\varphi }}_i|^2 \Big ) \nonumber \\&\ge (l-1)e_{\mathrm{cell}} + \frac{c_{\mathrm{conv}}}{ 4 (2l-1)} \Big (\sum _{i=1}^l |b_i - {\bar{b}}| + \sum _{i=2}^l |\varphi _i - {\bar{\varphi }}_i| \Big )^2. \end{aligned}$$

For $i=1,\ldots , l$ we let $\phi _i$ and ${\bar{\phi }}_i$ be the angles defined in (27), associated to *y* and ${\bar{y}}$, respectively. Possibly after rotations, it is not restrictive to suppose that $(y_{l+1} - y_1) \cdot e_2 = 0$ and that $\phi _1 = {\bar{\phi }}_1$. Clearly, we get $|\phi _i-{\bar{\phi }}_i| \le \sum _{j=2}^i |\varphi _j - {\bar{\varphi }}_j| \le \sum _{j=2}^l |\varphi _j - {\bar{\varphi }}_j|$ for all $i=2,\ldots ,l$. Since, $\cos$ is Lipschitz with constant 1, we then derive for each $i=1,\ldots ,l$

39 $$\begin{aligned} (y_{i+1} -y_i)\cdot e_1&= b_i \cos (\phi _i) \le {\bar{b}} \cos ({\phi }_i) + |b_i - {\bar{b}}| \le {\bar{b}} \cos ({\bar{\phi }}_i) + |b_i - {\bar{b}}| + {\bar{b}} |\phi _i-{\bar{\phi }}_i|\nonumber \\&\le {\bar{b}} \cos ({\bar{\phi }}_i) + |b_i - {\bar{b}}| + \sum _{j=2}^l |\varphi _j - {\bar{\varphi }}_j| \end{aligned}$$

where we also used the fact that ${\bar{b}}<1$. We now get

$$\begin{aligned} |y_{l+1}-y_1|&= \Big |\sum ^l_{i=1} (y_{i+1} -y_i)\cdot e_1\Big | \le \Big |\sum ^l_{i=1} {\bar{b}} \cos ({\bar{\phi }}_i)\Big | + \sum _{i=1}^l |b_i - {\bar{b}}| + l \sum _{i=2}^l |\varphi _i - {\bar{\varphi }}_i|\\&\le |{\bar{y}}_{l+1} - {\bar{y}}_1| + \sum _{i=1}^l |b_i - {\bar{b}}| + l \sum _{i=2}^l |\varphi _i - {\bar{\varphi }}_i| \end{aligned}$$

which together with (38) and the choice $C = c_{\mathrm{conv}}/( 4 (2l-1)l^2)$ gives the first inequality of the statement. The second inequality follows from Lemma 4.5. $\quad \square$

Similarly to Lemma 4.8, we now consider two consecutive waves with odd discrete-wave periods and provide a control on the length in terms of the junction angle.

#### Lemma 4.10

(Junction angle controls length excess) Let $\delta >0$ and $l_1,l_2 \in (2\mathbb {N}+1) \cap [2, l_{\mathrm{max}}]$. Let $y,{\bar{y}}: \lbrace 1,\ldots , l_1+l_2 + 1 \rbrace \rightarrow \mathbb {R}^2$ be configurations with

$$\begin{aligned}&y^1 := (y_1, \ldots ,y_{l_1+ 2 }) \in \mathcal {U}^{l_1 + 1 }_\delta , \ \ \ \ \ y^2:=(y_{l_1+ 2 } \ldots ,y_{l_1+l_2+1}) \in \mathcal {U}^{l_2 -1}_\delta ,\\&{\bar{y}}^1 := ({\bar{y}}_1, \ldots ,{\bar{y}}_{l_1 + 2 }) \in \mathcal {U}^{l_1 + 1 }, \ \ \ \ \ {\bar{y}}^2:=({\bar{y}}_{l_1+ 2 } \ldots ,{\bar{y}}_{l_1+l_2+1}) \in \mathcal {U}^{l_2 -1 } \end{aligned}$$

and ${\bar{y}}^i$, $i=1,2$, are configurations corresponding to $y^i$ as given in (36). Then we have

$$\begin{aligned} |y_{l_1+l_2+1} - y_1| \le |{\bar{y}}_{l_1+l_2+1} - {\bar{y}}_1| + 2l_{\mathrm{max}}| \varphi _{l_1+ 2 } -{\bar{\varphi }}_{l_1+ 2 }| + 4l^2_{\mathrm{max}}\delta . \end{aligned}$$

#### Proof

We denote the angles $\phi _i$ and ${\bar{\phi }}_i$ as in the previous proof, where the sum now runs from 1 to $l_1+l_2$. We may again suppose that, possibly after a rotation, we have $(y_{l_1+l_2+1} - y_1) \cdot e_2 = 0$ and that $\phi _1 = {\bar{\phi }}_1$. This implies $|\phi _i-{\bar{\phi }}_i| \le \sum _{j=2}^{l_1+l_2} |\varphi _j - {\bar{\varphi }}_j|$ for all $i =1,\ldots ,l_1+l_2$. Repeating the estimate in (39) and recalling (36) we find

$$\begin{aligned}&(y_{i+1} -y_i)\cdot e_1 - {\bar{b}} \cos ({\bar{\phi }}_i) \le |b_i - {\bar{b}}| \\&\quad + \sum _{j=2}^{l_1+l_2} |\varphi _j - {\bar{\varphi }}_j| \le \delta + (l_1 -1 + l_2-1)\delta + | \varphi _{l_1+ 2 } -{\bar{\varphi }}_{l_1+ 2}|. \end{aligned}$$

The claim follows by taking the sum over $i=1,\ldots ,l_1+l_2$. $\quad \square$

We close this section with the observation that also for configurations in $\mathcal {U}^l_{\delta }$ the maximal discrete-wave period is given by $l_{\mathrm{max}}$.

#### Lemma 4.11

(Maximal discrete-wave period). There exists $\delta _0 = \delta _0(\rho ) > 0$ such that for all $0 < \delta \le \delta _0$ we have $\mathcal {U}^l_\delta \cap \mathcal {A}_{l+1} = \emptyset$ for each $l \ge l_{\mathrm{max}}$.

#### Proof

We argue by contradiction. Suppose that there exists $y \in \mathcal {U}^l_\delta \cap \mathcal {A}_{l+1}$. Let ${\bar{y}} \in \mathcal {U}^l$ be an associated configuration from (36). As $l \ge l_{\mathrm{max}}$, we find $i_0 > \lceil 2\pi /{\bar{\psi }}\rceil -2$ or $l+1 - i_0 > \lceil 2\pi /{\bar{\psi }}\rceil -2$ with $i_0$ from (22). Possibly after inverting the labeling of the particles in the chain, we can assume that $i_0 \ge \lceil 2\pi /{\bar{\psi }}\rceil -1$. With $j = \lceil 2\pi /{\bar{\psi }} \rceil$, we repeat the proof of Lemma 4.1, see (25), to find $|{\bar{y}}_j - {\bar{y}}_1| \le 1$. Moreover, using (36) and adapting the argument leading to (39), we get

$$\begin{aligned} | ({\bar{y}}_j-{y}_j) - {({\bar{y}}_1-y_1)| } \le \sqrt{2} \big ( (j-1)\delta + (j-1)(l-1)\delta ). \end{aligned}$$

Consequently, for $\delta$ small enough depending only on $l_\mathrm{max}$ (and thus only on $\rho$, cf. Remark 3.2), we derive $|y_j - y_1| < 1.5$, which contradicts (1). $\quad \square$

## The Multiple-Period Problem

In this section, we study the relation between length and energy for a chain consisting of more than one single discrete-wave period. More precisely, we will investigate configurations belonging to

$$\begin{aligned}&\mathcal {A}^\delta _n:= \big \{ y\in \mathcal {A}_n \, | \ |b_i - {\bar{b}}| \le \delta , \ \\&\min \lbrace |\varphi _i - \pi -{\bar{\psi }}|, |\varphi _i - \pi +{\bar{\psi }}| \rbrace \le \delta \ \ \text {for all} \ i=1,\ldots ,n-1 \big \} \end{aligned}$$

for $\delta >0$ to be specified below, where the bond lengths $b_i$ and angles $\varphi _i$ are defined in (2). (As before, for the angles indices run only from 2 to $n-1$.) For later purpose, we note that by Lemma 3.1,(ii) we have

40 $$\begin{aligned} \frac{c_{\mathrm{conv}}}{ 4 } \Big (\sum _{i=1}^{n-1} |b_i - {\bar{b}}_i|^2 + \sum _{i=2}^{n-1} |\varphi _i - {\bar{\varphi }}_i|^2\Big ) \le E^\mathrm{red}_n(y) - (n-2) e_{\mathrm{cell}} \end{aligned}$$

for $c_{\mathrm{conv}} = c_{\mathrm{conv}}(\rho )>0$ and $\delta \le \delta _0$ with $\delta _0$ from Lemma 4.9, cf. (37) for the exact computation. We split our considerations into two parts concerning the reduced and the general energy, respectively.

### The multiple-period problem for the reduced energy

We introduce the index set

41 $$\begin{aligned} \mathcal {I}_{\mathrm{sgn}} = \lbrace i=3,\ldots ,n-2 \,| \ \varphi _{i} > \pi , \ \varphi _{i+1} < \pi \rbrace \cup \lbrace 1 \rbrace . \end{aligned}$$

The index set is denoted by ‘sgn’ to highlight that at the points $y_i$, $i \in \mathcal {I}_{\mathrm{sgn}}$, the sign of $\varphi _i- \pi$ changes from plus to minus. For the application in Sect. 6 it is convenient to also take the index $i=1$ into account. Sometimes we will also consider the ‘shifted’ index set

42 $$\begin{aligned} \mathcal {I}_{\mathrm{sgn}}' = \lbrace i=3,\ldots ,n-2 \,| \ \varphi _{i-1} > \pi , \ \varphi _{i} < \pi \rbrace . \end{aligned}$$

We also define a decomposition of $\mathcal {I}_{\mathrm{sgn}}$ by

43 $$\begin{aligned} \mathcal {I}^l_{\mathrm{sgn}} = \big \{ i \in \mathcal {I}_{\mathrm{sgn}} \,| \ i+k \notin \mathcal {I}_{\mathrm{sgn}} \text { for } k=1,\ldots ,l-1, \ i+l \in \mathcal {I}_{\mathrm{sgn}} \cup \lbrace n \rbrace \big \} \end{aligned}$$

for $$l \in \mathbb {N}$$, $$ l \ge 2$$.

For a minimizer *y* of $E^{\mathrm{red}}_n$, the length of the waves corresponding to even discrete-wave periods $(\mathcal {I}^l_\mathrm{sgn})_{l \in 2\mathbb {N}}$ can be estimated by

$$\begin{aligned} \sum _{l \in 2\mathbb {N}} \# \mathcal {I}_{\mathrm{sgn}}^l \lambda ^l_{\mathrm{max}} \le \sum _{l \in 2\mathbb {N}} \# \mathcal {I}_{\mathrm{sgn}}^l l\Lambda (l), \end{aligned}$$

where we used Lemma 4.5. In the previous section, see particularly Lemma 4.8, we have also seen that the length of waves with odd discrete-wave period can be controlled in terms of waves with even discrete-wave period. For later purpose, we introduce the *maximal length of odd discrete-wave periods* $\mathcal {L}: 2\mathbb {N}+ 1 \rightarrow (0,\infty )$ by

44 $$\begin{aligned} \mathcal {L}\big ( (\# \mathcal {I}^l_{\mathrm{sgn}})_{l \in 2\mathbb {N}+1 }\big )&= \max \Big \{ \sum _{l \in 2\mathbb {N}} r_l l\Lambda (l) \, \big | \ r_l \in \mathbb {N}\ \text { for } \ l \in 2\mathbb {N}: \nonumber \\&\quad \sum _{l \in 2\mathbb {N}} r_l = \sum _{l \in 2\mathbb {N}+1} \# \mathcal {I}^l_{\mathrm{sgn}}, \ \ \sum _{l \in 2\mathbb {N}} l r_l = 2\Big \lfloor \sum _{l \in 2\mathbb {N}+1} l \# \mathcal {I}^l_{\mathrm{sgn}}/2 \Big \rfloor \Big \}. \end{aligned}$$

Recall the definition of the maximal discrete-wave period $l_{\mathrm{max}}$ in (24). For convenience, we introduce also a relabeling $\mathcal {I}_{\mathrm{sgn}} \cup \lbrace n\rbrace = \lbrace i_1,\ldots ,i_{J} \rbrace$ for an increasing sequence of integers $(i_j)^J_{j=1}$. The following lemma controls (up to some boundary effects) the length of the chain in terms of the contributions of waves with even and odd discrete-wave periods.

#### Lemma 5.1

(Length of chain with minimal energy). Let $y : \lbrace 1,\ldots , n\rbrace \rightarrow \mathbb {R}^2$ with $y \in \mathcal {A}_n$ be a minimizer of $E^{\mathrm{red}}_n$. Then

$$\begin{aligned} |y_n-y_1| \le \sum _{l \in 2\mathbb {N}} \# \mathcal {I}_{\mathrm{sgn}}^l l\Lambda (l) + \mathcal {L}\big ( (\# \mathcal {I}^l_{\mathrm{sgn}})_{l \in 2\mathbb {N}+1 }\big ) - \frac{c_{\mathrm{mix}}}{2} \sum _{l \in 2\mathbb {N}+1} \# \mathcal {I}_{\mathrm{sgn}}^l + 4l_{\mathrm{max}}, \end{aligned}$$

where $c_{\mathrm{mix}}> 0$ is the constant from Lemma *4.8*.

#### Proof

Consider the labeling $$\mathcal {I}_{\mathrm{sgn}} \cup \lbrace n\rbrace = \lbrace i_1,\ldots ,i_{J} \rbrace $$. Moreover, we choose indices $$j_1< j_2< \ldots < j_K$$ such that $$\bigcup _{l \in 2\mathbb {N}+1} \mathcal {I}_{\mathrm{sgn}}^l = \lbrace i_{j_1},\ldots ,i_{j_K} \rbrace $$. Note that $$K=\sum _{l \in 2\mathbb {N}+1} \# \mathcal {I}_{\mathrm{sgn}}^l$$. In the following, we will consider pairs of indices $$i_{j_k}$$, $$i_{j_{k+1}}$$ for odd *k* with corresponding lengths $$l_1^k = i_{j_k+1} - i_{j_k} $$ and $$l_2^k = i_{j_{k+1}+1} - i_{j_{k+1}} $$. We will suppose that

45 $$\begin{aligned} \# \mathcal {K} \ge \lfloor K/2 \rfloor \ \ \ \text {with} \ \ \ \mathcal {K} := \lbrace k \,| \ l_1^k \ge l_2^k \rbrace . \end{aligned}$$

The case $\# \mathcal {K} < \lfloor K/2 \rfloor$ is very similar by considering the chain in reverse order. We indicate the necessary adaptions at the end of the proof.

Consider a pair of indices $$i_{j_k}$$, $$i_{j_{k+1}}$$ for odd *k*. Recall that $$l_1^k = i_{j_k+1} - i_{j_k} $$, $$l_2^k = i_{j_{k+1}+1} - i_{j_{k+1}} $$ are odd and $$ l_{m,k} := i_{j_k+m+1} - i_{j_k+m}$$ are even for all $$m \in \lbrace 1, \ldots , M_k-1 \rbrace $$, where $$M_k := j_{k+1} - j_k$$. We can decompose the chain $$(y_{i_{j_k}},\ldots , y_{i_{j_{k+1}+1}})$$ into the parts

46 $$\begin{aligned} {\hat{y}}^{0,k}&= (y_{i_{j_k}},\ldots ,y_{i_{j_k+1}+1}) \in \mathcal {U}^{l_1^k +1} \nonumber \\ {\hat{y}}^{m,k}&= (y_{i_{j_k+m}+1},\ldots ,y_{i_{j_k+m+1}+1}) \in \mathcal {U}^{l_{m,k}} \ \ \ \text {for} \ \ m \in \lbrace 1, \ldots , M_k-1 \rbrace , \nonumber \\ {\hat{y}}^{M_k,k}&= (y_{i_{j_{k+1}}+1},\ldots ,y_{i_{j_{k+1}+1}}) \in \mathcal {U}^{l_2^k -1}. \end{aligned}$$

Here, we have used Theorem 2.1 and the fact that $$ i_{j_k+m}+1 \in \mathcal {I}_{\mathrm{sgn}}'$$ (cf. (41)–(42)) to see that the chains have the form introduced in (21)–(22). (We refer to Fig. 8 for an illustration of composed single-period waves.) We also define the configuration $$ \tilde{y}^{k}: \lbrace l_1^k + l_2^k +1 \rbrace \rightarrow \mathbb {R}^2 $$ by (recall $$l_1^k = i_{j_k+1} - i_{j_k} $$ and $$l_2^k = i_{j_{k+1}+1} - i_{j_{k+1}} $$)

47 $$\begin{aligned} (\tilde{y}^{k}_1,\ldots , \tilde{y}^{k}_{l_1^k+2}) = {\hat{y}}^{0,k}, \ \ \ (\tilde{y}^{k}_{l_1^k+2},\ldots , \tilde{y}^{k}_{l^k_1+l_2^k+1}) = {\hat{y}}^{M_k,k} + {\varvec{t}} \end{aligned}$$

for the translation ${\varvec{t}} = (y_{i_{j_k+1}+1} - y_{i_{j_{k+1}}+1},\ldots ,y_{i_{j_k+1}+1} - y_{i_{j_{k+1}}+1}) \in \mathbb {R}^{2 \times l_2^k}$. By the definition of the function $\lambda ^{l}$ in (26) and the triangle inequality we get

48 $$\begin{aligned} |y_{i_{j_{k+1}+1}}- y_{i_{j_k}}|&\le |\tilde{y}^{k}_{l_1^k + l_2^k +1} - \tilde{y}^{k}_1| + \sum _{m=1}^{M_k-1} |{\hat{y}}^{m,k}_{l_{m,k}+1} - {\hat{y}}^m_1| \nonumber \\&\le |\tilde{y}^{k}_{l_1^k + l_2^k +1} - \tilde{y}^{k}_1| + \sum _{m=1}^{M_k-1} \lambda _{\mathrm{max}}^{l_{m,k}}. \end{aligned}$$

From Theorem 2.1 we recall that each angle $\varphi _i$ (see (2)) enclosed by two bonds is $\pi + {\bar{\psi }}$ or $\pi - {\bar{\psi }}$. Due to the fact that the discrete-wave periods $l_{m,k}$ for $m \in \lbrace 1,\ldots , M_k-1\rbrace$ are even, we find $(i_{j_{k+1}}+1) - (i_{j_k+1}+1) \in 2\mathbb {N}$. Thus,

$$\begin{aligned} \sphericalangle (y_{i_{j_{k+1}}+2} - y_{i_{j_{k+1}}+1}, y_{i_{j_k+1}+1} - y_{i_{j_k+1}} ) \in { \pi + } (1 + 2\mathbb {N}){\bar{\psi }}, \end{aligned}$$

i.e., the junction angle ${\tilde{\varphi }}_{l_1^k+2}$ at $\tilde{y}_{l_1^k+2}$ satisfies ${\tilde{\varphi }}_{l_1^k+2} - \pi \in (1 +2\mathbb {Z}){\bar{\psi }}$. Consequently, we can apply Lemma 4.8 and find together with (48)

49 $$\begin{aligned} |y_{i_{j_{k+1}+1}} - y_{i_{j_k}}|&\le \max _{t \in \lbrace -1,1\rbrace } \big ( (l_1^k + t)\Lambda (l_1^k + t) +(l_2^k - t)\Lambda (l_2^k - t) \big ) \nonumber \\&\quad -c_\mathrm{mix}{\varvec{1}}_{\mathcal {K}}(k) + \sum _{m=1}^{M_k-1} l_{m,k}\Lambda (l_{m,k}). \end{aligned}$$

Here, we have also used that the discrete-wave periods $l_1^k+t$, $l_2^k-t$, and $l_{m,k}$ are even and have applied Lemma 4.5.

We proceed in this way for all $k \in \lbrace 1,3,\ldots , 2\lfloor K/2\rfloor -1 \rbrace$ and then we derive by (45), (49), Lemma 4.5, and the triangle inequality

$$\begin{aligned} |y_n-y_1|&\le \sum _{l \in 2\mathbb {N}} \#\mathcal {I}_{\mathrm{sgn}}^l l\Lambda (l) + \sum _{ k \, \mathrm{odd} } \max _{t \in \lbrace -1,1\rbrace }\\&\quad \big ( (l_1^k + t)\Lambda (l_1^k + t) +(l_2^k - t)\Lambda (l_2^k - t) \big ) - \lfloor K/2 \rfloor c_{\mathrm{mix}} \nonumber \\&\quad + |y_{i_2}-y_1| + |y_n - y_{i_{J-1}}| + |y_{i_{j_K+1}} - y_{i_{j_K}} |, \end{aligned}$$

where here and in the following the sum in *k* always runs over $\lbrace 1,3,\ldots , 2\lfloor K/2\rfloor -1 \rbrace$. Note that the last three terms appear since Lemma 4.5 and the estimate (49) are possibly not applicable. (The very last term is only necessary for odd *K*.) However, in view of $y \in \mathcal {A}_n$, Lemma 4.1, and the fact that ${\bar{b}} \le 1$ (see Theorem 2.1), their contribution can be bounded by $3l_{\mathrm{max}}$. Moreover, note that $\lfloor K/2 \rfloor \ge \sum _{l \in 2\mathbb {N}+1} \# \mathcal {I}_{\mathrm{sgn}}^l/2 -1$ and $c_{\mathrm{mix}} \in (0,1)$. To conclude, it therefore remains to show

50 $$\begin{aligned} \sum _{ k \, \mathrm{odd} } \max _{t \in \lbrace -1,1\rbrace } \big ( (l_1^k + t)\Lambda (l_1^k + t) +(l_2^k - t)\Lambda (l_2^k - t) \big )\le \mathcal {L}\big ( (\# \mathcal {I}^l_{\mathrm{sgn}})_{l \in 2\mathbb {N}+1 }\big ). \end{aligned}$$

For each *k* choose $t_k \in \lbrace -1,1 \rbrace$ such that the maximum is attained. If *K* is even, we set $r_l = \# \lbrace k \, | \ l_1^k + t_k = l \rbrace + \# \lbrace k \, | \ l_2^k - t_k = l \rbrace$ for $l \in 2\mathbb {N}$. If *K* is odd, we replace $r_t$ by $r_t + 1$, where $t = i_{j_K+1} - i_{j_K} -1 \in 2\mathbb {N}$. We then find $\sum _{l\in 2\mathbb {N}} r_l = K =\sum _{l \in 2\mathbb {N}+1} \# \mathcal {I}_\mathrm{sgn}^l$ and $\sum _{l\in 2\mathbb {N}} lr_l = 2 \lfloor \sum _{l \in 2\mathbb {N}+1} l \# \mathcal {I}^l_{\mathrm{sgn}}/2 \rfloor$. Then (50) follows from (44).

Finally, we briefly indicate the necessary changes if $K - \# \mathcal {K} \ge \lceil K/2 \rceil$ (see (45)). In this case, we consider the chain ${\hat{y}} = (y_n,\ldots ,y_1)$ in reverse order and note that the index set introduced in (41) corresponding to ${\hat{y}}$ is given by $\mathcal {I}_{\mathrm{sgn}}' \cup \lbrace n \rbrace$ (as defined in (42) for the configuration *y*). The above reasoning is then applied on the pairs of indices $i_{j_{k+1}} + 1$ and $i_{j_k}+1$ for $k \in \lbrace 1,3,\ldots , 2\lfloor K/2\rfloor -1 \rbrace$, where we note that $\# \lbrace k \, | \ i_{j_{k+1}+1} -i_{j_{k+1}} \ge i_{j_k+1} - i_{j_k}\rbrace \ge \lceil K/2 \rceil \ge \lfloor K/2 \rfloor$. $\quad \square$

We now investigate the length of general configurations in $\mathcal {A}^\delta _n$. Recall the notation $(a)_+^2 = (\max \lbrace a,0\rbrace )^2$ for $a \in \mathbb {R}$.

#### Lemma 5.2

(Energy excess controls length excess) There exist $\delta _0>0$, $c_{\mathrm{odd}}>0$, and $c_{\mathrm{el}}>0$ only depending on $\rho$ such that for all $0 \le \delta \le \delta _0$ and each $y \in \mathcal {A}^\delta _n$ we have

$$\begin{aligned} E^{\mathrm{red}}_n(y) - (n-2)e_{\mathrm{cell}}\ge & {} \frac{c_{\mathrm{el}} }{n} \Big (|y_n-y_1| - \sum _{l \in 2\mathbb {N}} \# \mathcal {I}_{\mathrm{sgn}}^l l\Lambda (l) \\&-\mathcal {L}\big ( (\# \mathcal {I}^l_{\mathrm{sgn}})_{l \in 2\mathbb {N}+1 }\big ) + n_{\mathrm{odd}}c_{\mathrm{odd}} - 4 l_{\mathrm{max}} \Big )^2_+, \end{aligned}$$

where $\mathcal {I}_{\mathrm{sgn}}^l$ as in (43) and $n_{\mathrm{odd}} = \sum _{l \in 2\mathbb {N}+1} \# \mathcal {I}_{\mathrm{sgn}}^l l$.

#### Proof

Let $y \in \mathcal {A}^\delta _n$ be given and define $\mathcal {I}_{\mathrm{sgn}}$ and $\mathcal {I}_{\mathrm{sgn}}^l$ as in (41) and (43), respectively. Choose a configuration ${\bar{y}}: \lbrace 1,\ldots , n\rbrace \rightarrow \mathbb {R}^2$ minimizing the energy $E^{\mathrm{red}}_n$ and satisfying $\mathrm{sgn}(\varphi _i - \pi ) = \mathrm{sgn}({\bar{\varphi }}_{i} - \pi )$ for $i=2,\ldots ,n-1$, where $\mathrm{sgn}$ denotes the sign function and ${\bar{\varphi }}_i$ are the angles defined in (2) corresponding to ${\bar{y}}$. Note that ${\bar{y}}$ is determined uniquely by *y* up to a rigid motion.

We will follow the lines of the previous proof by taking additionally the deviation from energy minimizers into account, where we will employ Lemma 4.9 and Lemma 4.10. Similarly to the previous proof, we consider the labeling $$\mathcal {I}_{\mathrm{sgn}} \cup \lbrace n\rbrace = \lbrace i_1,\ldots ,i_{J} \rbrace $$ as well as $$\bigcup _{l \in 2\mathbb {N}+1} \mathcal {I}_{\mathrm{sgn}}^l = \lbrace i_{j_1},\ldots ,i_{j_K} \rbrace $$. For odd *k* we also define $$l_1^k = i_{j_k+1} - i_{j_k} $$ and $$l_2^k = i_{j_{k+1}+1} - i_{j_{k+1}} $$. Moreover, let $$\mathcal {K}$$ be defined as in (45). Without loss of generality we can reduce ourselves to the case $$\# \mathcal {K} \ge \lfloor K/2 \rfloor $$ since otherwise one may consider the chain in reverse order, as commented at the end of the previous proof.

We consider the parts of the chain having odd discrete-wave period. For odd *k*, we define the configuration $\tilde{y}^{k}: \lbrace 1,\dots , l_1^k + l_2^k +1 \rbrace \rightarrow \mathbb {R}^2$ as in (47). Accordingly, we define the configuration $\tilde{{\bar{y}}}^k$ corresponding to ${\bar{y}}$. By the triangle inequality (cf. (48)) we get that

51 $$\begin{aligned} |y_{i_{j_{k+1}+1}}- y_{i_{j_k}}| \le |\tilde{y}^{k}_{l_1^k + l_2^k +1} - \tilde{y}^{k}_1| + \sum _{m=1}^{M_k-1} |{\hat{y}}^{m,k}_{l_{m,k}+1} - {\hat{y}}^m_1|, \end{aligned}$$

where ${\hat{y}}^{m,k}$ is defined in (46). We now estimate the various terms in the above right-hand side by starting with the terms including ${\hat{y}}^{m,k}$. By Lemma 4.9, Hölder’s inequality, and the fact that $\sum _k (M_k-1) \le \sum _{l \in 2\mathbb {N}}\# \mathcal {I}_{\mathrm{sgn}}^l \le n$ we obtain

52 $$\begin{aligned}&\sum _{k \, \mathrm{odd}} \sum _{m=1}^{M_k-1} ( |{\hat{y}}^{m,k}_{l_{m,k}+1} - {\hat{y}}^m_1| - l_{m,k}\Lambda (l_{m,k})) \nonumber \\&\le \frac{1}{\sqrt{C}} \sum _{k \, \mathrm{odd}} \sum _{m=1}^{M_k-1} \big (E^{\mathrm{red}}_{l_{m,k}+1}({\hat{y}}_{m,k}) - (l_{m,k}-1)e_{\mathrm{cell}}\big )^{1/2} \nonumber \\&\le { \frac{\sqrt{n}}{\sqrt{C}} \Big (\sum _{k \, \mathrm{odd}} \sum _{m=1}^{M_k-1} \big (E^{\mathrm{red}}_{l_{m,k}+1}({\hat{y}}_{m,k}) - (l_{m,k}-1)e_{\mathrm{cell}}\big ) \Big )^{1/2} } \nonumber \\&\le \frac{ \sqrt{n}}{\sqrt{C}} \big (E^{\mathrm{red}}_n(y) - (n-2)c_{\mathrm{cell}} \big )^{1/2} \end{aligned}$$

with $C>0$ from Lemma 4.9, where in the last step we have used Theorem 2.1.

We now consider the first term in the right-hand side of (51). The difference of the junction angles $\tilde{{\varphi }}^k_{l_1^k+ 2 }$ and $\tilde{{\bar{\varphi }}}^k_{l_1^k+ 2 }$ at $\tilde{y}^k_{l_1^k+ 2 }$ and $\tilde{{\bar{y}}}^k_{l_1^k+ 2 }$, respectively, can be estimated by

$$\begin{aligned} |\tilde{{\varphi }}_{l_1^k+ 2 } - \tilde{{\bar{\varphi }}}_{l_1^k+ 2 }| \le \sum _{i=i_{j_k+1} { + 1 } }^{i_{j_{k+1}} { + 1 } } |\varphi _i - {\bar{\varphi }}_i|. \end{aligned}$$

Consequently, applying Lemma 4.10 and summing over all $k \in \lbrace 1,3,\ldots , 2\lfloor K/2\rfloor -1 \rbrace$ we derive

$$\begin{aligned} \sum _{k \, \mathrm{odd} } (|\tilde{y}^k_{l_1^k + l_2^k +1} - \tilde{y}_1^k| - |\tilde{{\bar{y}}}^k_{l_1^k + l_2^k +1} - \tilde{{\bar{y}}}_1^k|)&\le 2l^2_{\mathrm{max}} K\delta + 2l_{\mathrm{max}} \sum _{i=2}^{n-1} |\varphi _i - {\bar{\varphi }}_i|. \end{aligned}$$

Repeating the arguments in (48)–(50), in particular using Lemma 4.8 for $|\tilde{{\bar{y}}}^k_{l_1^k + l_2^k +1} - \tilde{{\bar{y}}}_1^k|$, we find

$$\begin{aligned} \sum _{k \, \mathrm{odd}} |\tilde{y}^k_{l_1^k + l_2^k +1} - \tilde{y}_1^k| \le \mathcal {L}\big ( (\# \mathcal {I}^l_\mathrm{sgn})_{l \in 2\mathbb {N}+1 }\big ) - \lfloor K/2 \rfloor c_{\mathrm{mix}} + 2l^2_{\mathrm{max}} K\delta + 2l_{\mathrm{max}} \sum _{i=2}^{n-1} |\varphi _i - {\bar{\varphi }}_i|. \end{aligned}$$

For brevity we set $E = E^{\mathrm{red}}_n(y) - (n-2)e_{\mathrm{cell}}$. Combining the previous estimate with (51) and (52), and using again Hölder’s inequality together with (40), we get

$$\begin{aligned} \sum _{k \, \mathrm{odd} } |y_{i_{j_{k+1}+1}}- y_{i_{j_k}}|&\le \mathcal {L}\big ( (\# \mathcal {I}^l_{\mathrm{sgn}})_{l \in 2\mathbb {N}+1 }\big ) + \sum _{k \, \mathrm{odd} } \sum _{m=1}^{M_k-1} l_{m,k}\Lambda (l_{m,k}) - \lfloor K/2 \rfloor c_{\mathrm{mix}} \nonumber \\&\ \ \ + 2l^2_{\mathrm{max}} K\delta + \Big ( \frac{1}{\sqrt{C}} + \frac{4 l_{\mathrm{max}}}{\sqrt{c_{\mathrm{conv}}}} \Big )\sqrt{n} \sqrt{E}. \end{aligned}$$

For the remaining parts with even period we repeat the argument in (52). All in all we get

$$\begin{aligned} |y_n-y_1|&\le \sum _{l \in 2\mathbb {N}} \# \mathcal {I}_{\mathrm{sgn}}^l l\Lambda (l) + \mathcal {L}\big ( (\# \mathcal {I}^l_{\mathrm{sgn}})_{l \in 2\mathbb {N}+1 }\big ) - \lfloor K/2 \rfloor c_{\mathrm{mix}} + 2l^2_\mathrm{max} K\delta \\&\ \ \ + \Big ( \frac{2}{\sqrt{C}} + \frac{4 l_{\mathrm{max}}}{\sqrt{c_{\mathrm{conv}}}} \Big )\sqrt{n} \sqrt{E} + |y_{i_2}-y_1| + |y_n - y_{i_{J-1}}| + |y_{i_{j_K+1}} - y_{i_{j_K}} |, \end{aligned}$$

where the last three terms appear since Lemma 4.9 is possibly not applicable on these parts of the chain. (The very last term is only necessary for odd *K*.) Similarly to the proof of Lemma 5.1, by Lemma 4.11 we can show that $|y_{i_2}-y_1| + |y_n - y_{i_{J-1}}| + |y_{i_{j_K+1}} - y_{i_{j_K}} | \le 3({\bar{b}} + \delta )l_{\mathrm{max}} \le 3l_{\mathrm{max}}$ for $\delta _0$ sufficiently small. Therefore, using also $\lfloor K/2 \rfloor \ge \sum _{l \in 2\mathbb {N}+1} \# \mathcal {I}_{\mathrm{sgn}}^l/2 -1$ and $c_{\mathrm{mix}} \in (0,1)$ we get

$$\begin{aligned} |y_n-y_1|&\le \sum _{l \in 2\mathbb {N}} \# \mathcal {I}_{\mathrm{sgn}}^l l\Lambda (l) + \mathcal {L}\big ( (\# \mathcal {I}^l_{\mathrm{sgn}})_{l \in 2\mathbb {N}+1 }\big ) - \frac{c_{\mathrm{mix}}}{2} \sum _{l \in 2\mathbb {N}+1} \# \mathcal {I}_{\mathrm{sgn}}^l + 4 l_{\mathrm{max}} + 2l^2_{\mathrm{max}} K\delta \\&\ \ \ + \frac{\sqrt{n}}{\sqrt{c_{\mathrm{el}}}} \big (E^\mathrm{red}_n(y) - (n-2)c_{\mathrm{cell}}\big )^{1/2}, \end{aligned}$$

where $c_{\mathrm{el}} := ( 2 / \sqrt{C} + 4 l_\mathrm{max}/\sqrt{c_{\mathrm{conv}}})^{-2}$. Now choose $\delta _0$ so small that $2l^2_{\mathrm{max}} \delta \le c_{\mathrm{mix}}/4$ and set $c_{\mathrm{odd}} = c_{\mathrm{mix}}/(4l_{\mathrm{max}})$. This implies $(c_{\mathrm{mix}}/2 - 2l^2_{\mathrm{max}} \delta ) K\ge c_{\mathrm{odd}} l_{\mathrm{max}} K \ge c_\mathrm{odd} n_{\mathrm{odd}}$, where the last step follows from $n_\mathrm{odd}/l_{\mathrm{max}} \le \sum _{l \in 2\mathbb {N}+1} \# \mathcal {I}_{\mathrm{sgn}}^l = K$. From this, the statement of the lemma follows. $\quad \square$

### The multiple-period problem for the general energy

Let $y \in \mathcal {A}_n$ and observe that the general energy (7) including the longer-range interaction can be written as

53 $$\begin{aligned} E_n (y) = \sum _{i=1}^{n-3} E^{\mathrm{gen}}_{\mathrm{cell}} (y_i, y_{i+1},y_{i+2},y_{i+3}) +\frac{1}{2}\big (E_\mathrm{cell}(y_{1},y_{2},y_{3}) +E_{\mathrm{cell}}(y_{n-2},y_{n-1},y_{n})\big ), \end{aligned}$$

where $E^{\mathrm{gen}}_{\mathrm{cell}}$ denotes the *generalized cell energy* defined by

$$\begin{aligned} E^{\mathrm{gen}}_{\mathrm{cell}} (y_i, y_{i+1},y_{i+2},y_{i+3})&= \frac{1}{2}\big ( E_{\mathrm{cell}}(y_{i}, y_{i+1},y_{i+2})&+ E_\mathrm{cell}(y_{i+1},y_{i+2},y_{i+3}) \big ) \\&\quad + {\bar{\rho }}v_2(|y_{i+3} - y_i|). \end{aligned}$$

Let $y \in \mathcal {A}_n$ be a minimizer of $E^{\mathrm{red}}_n$. Choose $i \in \lbrace 1,\ldots ,n-3\rbrace$ with $\mathrm{sgn}(\varphi _{i+1}-\pi ) = \mathrm{sgn}(\varphi _{i+2}-\pi )$, where $\mathrm{sgn}$ denotes the sign function, and define

54 $$\begin{aligned} e^{\mathrm{gen}}_{\mathrm{cell}} := E^{\mathrm{gen}}_{\mathrm{cell}} (y_i, y_{i+1},y_{i+2},y_{i+3}). \end{aligned}$$

Clearly, the value is independent of the particular choice of the configuration *y* and the index *i*. Recalling Theorem 2.1, we also see $|e^{\mathrm{gen}}_{\mathrm{cell}} - e_{\mathrm{cell}}| \le c{\bar{\rho }}$ for some $c>0$. Now choose $i \in \lbrace 1,\ldots ,n-3\rbrace$ with $\mathrm{sgn}(\varphi _{i+1}-\pi ) \ne \mathrm{sgn}(\varphi _{i+2}-\pi )$ and define

55 $$\begin{aligned} e_{\mathrm{per}} := \big ( E^{\mathrm{gen}}_{\mathrm{cell}} (y_i, y_{i+1},y_{i+2},y_{i+3}) - e^{\mathrm{gen}}_{\mathrm{cell}}\big )/{\bar{\rho }}. \end{aligned}$$

**Fig. 9:**
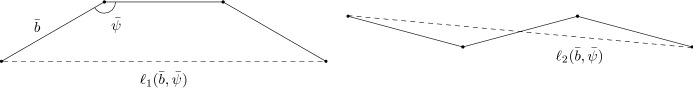
Two different geometries of four points with $l_1({\bar{b}},{\bar{\psi }}) < l_2({\bar{b}},{\bar{\psi }})$

As before, the value is independent of *y* and the choice of *i*. We find $e_{\mathrm{per}}>0$, which follows from the geometry of the four points $y_i,y_{i+1},y_{i+2},y_{i+3}$ determined by the condition $\mathrm{sgn}(\varphi _{i+1}-\pi ) = \mathrm{sgn}(\varphi _{i+2}-\pi )$ and $\mathrm{sgn}(\varphi _{i+1}-\pi ) \ne \mathrm{sgn}(\varphi _{i+2}-\pi )$, respectively, and the fact that $v_2$ is strictly increasing right of 1. (We refer to Fig. 9 for an illustration.)

Recall the definition of $\mathcal {I}_{\mathrm{sgn}}$ in (41). The general energy (53) for a configuration $y \in \mathcal {A}_n$ with $E^{\mathrm{red}}_n(y) = (n-2)e_{\mathrm{cell}}$ can now be estimated by

56 $$\begin{aligned} E_n(y) \ge (n-3) e^{\mathrm{gen}}_{\mathrm{cell}} + e_{\mathrm{cell}} + (2 \# \mathcal {I}_{\mathrm{sgn}}- 3 ) {\bar{\rho }} e_{\mathrm{per}}, \end{aligned}$$

where we used that $\# \lbrace i = 1,\ldots ,n-3 \,| \ \mathrm{sgn}(\varphi _{i+1}-\pi ) \ne \mathrm{sgn}(\varphi _{i+2}-\pi ) \rbrace \ge 2 \# \mathcal {I}_{\mathrm{sgn}}- 3$.

We now formulate the main result of this section about the relation between the reduced and the general energy.

#### Lemma 5.3

(Relation of reduced and general energy). There exist $\delta _0>0$ and $c_{\mathrm{range}}>0$ only depending on $\rho$ such that for all $0 \le \delta \le \delta _0$ and each $y \in \mathcal {A}^\delta _n$ we get

$$\begin{aligned} \frac{1}{2}\big (E_n^{\mathrm{red}}(y) - (n-2)e_{\mathrm{cell}}\big ) \le E_n(y) - \big ( (n-3)e^{\mathrm{gen}}_{\mathrm{cell}} + e_{\mathrm{cell}} + (2 \# \mathcal {I}_{\mathrm{sgn}}- 3 ) {\bar{\rho }} e_{\mathrm{per}} \big ) + nc_\mathrm{range} {\bar{\rho }}^2. \end{aligned}$$

Notice that the higher order error term $n c_{\mathrm{range}} {\bar{\rho }}^2$ appears since due to the longer-range interactions, the energy can be slightly decreased by small rearrangement of the particles. Note that Lemma 5.3 together with Lemma 5.2 allows to control the length excess in terms of the energy excess for the general energy.

#### Proof

Let $y \in \mathcal {A}^\delta _n$ be given. Exactly as in the proof of Lemma 5.2, we choose a configuration ${\bar{y}}: \lbrace 1,\ldots , n\rbrace \rightarrow \mathbb {R}^2$ minimizing the energy $E^{\mathrm{red}}_n$ and satisfying $\mathrm{sgn}(\varphi _i - \pi ) = \mathrm{sgn}({\bar{\varphi }}_{i} - \pi )$ for $i=2,\ldots ,n-1$, where $\varphi _i, {\bar{\varphi }}_i$ are the angles defined in (2) corresponding to *y* and ${\bar{y}}$, respectively. Denote the bonds introduced in (2) again by $b_i$ and ${\bar{b}}_i$. Recall that the energy $E_n({\bar{y}})$ can be estimated by (56). Using (40) and Theorem 2.1 we find

$$\begin{aligned} E:= E_n^{\mathrm{red}}(y) - (n-2)e_{\mathrm{cell}}= & {} E^{\mathrm{red}}_n(y) - E^{\mathrm{red}}_n({\bar{y}}) \ge \frac{c_{\mathrm{conv}}}{ 8 } \Big (\sum _{i=1}^{n-1} |b_i - {\bar{b}}_i|^2\\&\quad + \sum _{i=2}^{n-1} |\varphi _i - {\bar{\varphi }}_i|^2\Big ) + \frac{E}{2}. \end{aligned}$$

For each $i\in \lbrace 1,\ldots ,n-3\rbrace$ an elementary computation shows

$$\begin{aligned}&\big ||y_{i+3} - y_i| - |{\bar{y}}_{i+3}-{\bar{y}}_i|\big | \le c\big (|b_i - {\bar{b}}_i|+|b_{i+1} - {\bar{b}}_{i+1}|+|b_{i+2} - {\bar{b}}_{i+2}| \\&\quad + |\varphi _{i+1} - {\bar{\varphi }}_{i+1}|+ |\varphi _{i+2} - {\bar{\varphi }}_{i+2}| \big ) \end{aligned}$$

for some $c>0$. (The argument is very similar to the one in (39) and we therefore omit the details.) Consequently, we find a constant ${\bar{C}}>0$ only depending on $v_2'$ such that

$$\begin{aligned} E_n(y) - E_n({\bar{y}})&\ge E^{\mathrm{red}}_n(y) - E^{\mathrm{red}}_n({\bar{y}}) - {\bar{C}}{\bar{\rho }}\Big (\sum _{i=1}^{n-1} |b_i - {\bar{b}}_i| + \sum _{i=2}^{n-1}|\varphi _i-{\bar{\varphi }}_i| \Big ) \\&\ge \frac{c_{\mathrm{conv}}}{ 8 } \Big (\sum _{i=1}^{n-1} |b_i - {\bar{b}}_i|^2+ \sum _{i=2}^{n-1} |\varphi _i - {\bar{\varphi }}_i|^2\Big )\\&\quad + \frac{E}{2} - {\bar{C}}{\bar{\rho }}\Big (\sum _{i=1}^{n-1} |b_i - {\bar{b}}_i| + \sum _{i=2}^{n-1}|\varphi _i-{\bar{\varphi }}_i| \Big ). \end{aligned}$$

Minimizing the last expression amounts to choosing each $|b_i - {\bar{b}}_i|$ and $|\varphi _i-{\bar{\varphi }}_i|,$ equal to $4 {\bar{C}}{\bar{\rho }}/c_{\mathrm{conv}}$. Thus, we deduce

$$\begin{aligned} E_n(y) - E_n({\bar{y}})&\ge -(2n-3) 2 {\bar{C}}^2{\bar{\rho }}^2/c_\mathrm{conv} + E/2. \end{aligned}$$

This together with (56) yields the claim for $c_{\mathrm{range}} = 4{\bar{C}}^2/c_{\mathrm{conv}}$. $\quad \square$

## Proof of the Main Result

In this section we give the proofs of our main results Theorems 2.2–2.4. We firstly treat the upper bound for the minimal energy. Afterwards, we tackle the lower bound and the characterization of the almost minimizers.

Let us first define the function $e_{\mathrm{range}}$ being the main object of Theorem 2.2. We introduce the mapping $\Upsilon : [2,\infty ) \rightarrow \infty$ by defining it on even periods as

57 $$\begin{aligned} \Upsilon (l) = 2/l \ \ \ \ \text {for} \ \ \ \ l \in 2\mathbb {N}\end{aligned}$$

and making it affine on $[l,l+2], l \in 2\mathbb {N}$. (Similarly to the definition of $\Lambda$ in (30), the fact that the function is piecewise affine is crucial for the characterization of minimizers in Theorem 2.4.) Let $M \subset (2/3,1)$ be the closed interval introduced right before (6). We now define the function $e_{\mathrm{range}}: M \rightarrow \mathbb {R}$ by

58 $$\begin{aligned} e_{\mathrm{range}}(\mu ) = e_{\mathrm{per}}\Upsilon (\Lambda ^{-1}(\mu )) \end{aligned}$$

where the constant $e_{\mathrm{per}}$ comes from (55). First, note that $e_{\mathrm{range}}$ is well defined. Indeed, $\Lambda ^{-1}$ exists due to the strict monotonicity of $\Lambda$ and the image satisfies $\Lambda ([2,l_{\mathrm{mid}}]) \supset M$ for $\rho$ and thus ${\bar{\psi }}$ sufficiently small (see Lemma 4.5 and recall that $l_{\mathrm{mid}} = \lfloor 6/{\bar{\psi }} \rfloor$). Clearly, $e_{\mathrm{range}}$ is piecewise affine since $\Lambda$ and $\Upsilon$ are piecewise affine. More precisely, in view of (30), the points where $e_{\mathrm{range}}$ is not differentiable are given by $\lbrace \mu =\Lambda (l)| \ l \in 2 \mathbb {N}\cap \Lambda ^{-1}(M)\rbrace$. Recall that this set is denoted by $M_{\mathrm{res}}$, cf. (10). Moreover, recall that the values are given explicitly by formula (15), see also Lemma 4.3. Finally, the properties that $e_{\mathrm{range}}$ is increasing and convex follow from the facts that $\Upsilon$ is decreasing and convex, and $\Lambda ^{-1}$ is decreasing and concave.

**Fig. 10:**
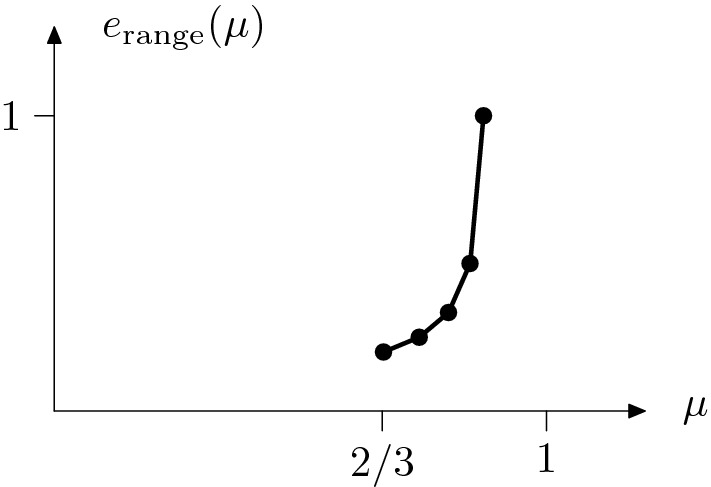
The function $$e_\mathrm{range}$$

### Upper bound for the minimal energy

Let us now address the upper bound for $E_{\mathrm{min}}^{n,\mu }$ in Theorem 2.2.

#### Proof of Theorem 2.2, upper bound

We first suppose that $\mu \in M_{\mathrm{res}}$, i.e., we find $l \in 2\mathbb {N}\cap [2,l_{\mathrm{mid}}]$ with $\mu = \Lambda (l)$. Let $y^{\mathrm{max}, l} \in \mathcal {U}^l$ be the configuration from Remark 4.7, see (31). Now we consider the configuration ${\bar{y}} \in \mathcal {A}_n$ defined by

$$\begin{aligned} {\bar{y}}_{i} = y^{\mathrm{max}, l}_{1 + ( i-1) \mathrm{mod}l} + \lfloor (i-1)/l \rfloor l\Lambda (l) e_1 \ \ \ \text {for} \ 1 \le i \le n. \end{aligned}$$

Similarly to Remark 4.7 and Fig. 8, recalling (21)–(22), we find that all bonds and angles of ${\bar{y}}$ (see (2)) satisfy $b_i = {\bar{b}}$ and ${{{\bar{\varphi }}}}_i = \pi \pm {\bar{\psi }}$. Thus, $E^\mathrm{red}_{n}({\bar{y}}) = (n-2)e_{\mathrm{cell}}$. By counting the number of indices with $\mathrm{sgn}({\bar{\varphi }}_{i} - \pi ) \ne \mathrm{sgn}({\bar{\varphi }}_{i+1} - \pi )$, similarly to (56) we deduce

59 $$\begin{aligned} E_{n}({\bar{y}})&\le (n-3) e^{\mathrm{gen}}_{\mathrm{cell}} + e_{\mathrm{cell}} + 2\lceil n/ l \rceil {\bar{\rho }} e_{\mathrm{per}}\nonumber \\&\le n \big (e^\mathrm{gen}_{\mathrm{cell}} + {\bar{\rho }} e_{\mathrm{range}}(\mu ) \big ) + c \end{aligned}$$

for a constant $c=c(\rho )>0$, where we used that $2e_{\mathrm{per}}/l = e_{\mathrm{per}}\Upsilon (l) = e_{\mathrm{range}}(\mu )$, see (57)–(58). Note that ${\bar{y}}$ possibly does not satisfy the boundary conditions if $n-1$ is not an integer multiple of *l*. However, in view of the fact that ${\bar{b}} \le 1$, $\mu =\Lambda (l) \le 1$ and $l \le l_{\mathrm{max}}$, we have $||{\bar{y}}_n - {\bar{y}}_1| - (n-1)\mu |\le 2l_{\mathrm{max}}$. Let $\varepsilon = ((n-1)\mu - |{\bar{y}}_n-{\bar{y}}_1|)/ |{\bar{y}}_n-{\bar{y}}_1|$ and note that $y:=(1+ \varepsilon ){\bar{y}} \in \mathcal {A}_n(\mu )$. It is not restrictive to suppose that $n \ge 8l_{\mathrm{max}}$ as otherwise (9) holds trivially. In that case, we find $\varepsilon \in (-4l_{\mathrm{max}}/(n\mu ),4l_{\mathrm{max}}/(n\mu ))$ after a short computation. Moreover, recalling the definition of the energy in (7) and the fact that $\tilde{E}_{\mathrm{cell}}$ grows quadratically around $({\bar{b}},{\bar{b}}, \pi \pm {\bar{\psi }})$, we obtain

$$\begin{aligned} E_n(y) \le E_n({\bar{y}}) + c n (\varepsilon ^2 + {\bar{\rho }}\varepsilon ) \end{aligned}$$

for $c=c(\rho )>0$. Thus, recalling the estimate for $\varepsilon$ and (59), the minimal energy $E^{n,\mu }_{\mathrm{min}}$ introduced in (8) satisfies $E^{n,\mu }_{\mathrm{min}} \le E_n(y)/(n-2) \le e^\mathrm{gen}_{\mathrm{cell}} + {\bar{\rho }} e_{\mathrm{range}}(\mu ) + c/n$.

We now move on to the general case $\mu \in M$. Choose $\mu ', \mu '' \in M_{\mathrm{res}}$ such that $(\mu ',\mu '') \cap M_{\mathrm{res}} = \emptyset$ and $\mu = \nu \mu ' + (1- \nu )\mu ''$ for some $\nu \in [0,1]$. Moreover, let $l' \in 2\mathbb {N}$ such that $\mu ' = \Lambda (l')$ and $\mu '' = \Lambda (l'')$, where $l'' = l' + 2 \in [2,l_\mathrm{mid}]$. For brevity, we set $N = l'\lfloor \nu n /l'\rfloor$ and consider the configuration ${\bar{y}} \in \mathcal {A}_n$ defined by

60 $$\begin{aligned} {\bar{y}}_{i}&= y^{\mathrm{max}, l'}_{1 + ( i-1) \mathrm{mod}l'} + \lfloor (i-1)/l' \rfloor l'\Lambda (l') e_1, \ \ \ \ \ \ \ \ \ \ \text {for} \ i \le N,\nonumber \\ {\bar{y}}_{i}&= N \Lambda (l') e_1 + y^{\mathrm{max}, l''}_{1 + ( i- N-1) \mathrm{mod}l''} + \lfloor (i-N-1)/l'' \rfloor l''\Lambda (l'') e_1 \ \ \ \text {for} \ i > N \end{aligned}$$

for $y^{\mathrm{max}, l'}$ and $y^{\mathrm{max}, l''}$ as introduced in (31). As before, we obtain $E^{\mathrm{red}}_{n}({\bar{y}}) = (n-2)e_{\mathrm{cell}} + c$ for some $c=c(\rho )>0$. Here, the extra term is due to the fact that $E_{\mathrm{cell}}({\bar{y}}_N, {\bar{y}}_{N+1},{\bar{y}}_{N+2}) > e_{\mathrm{cell}}$ since ${\bar{\varphi }}_{N+1} \ne \pi \pm {\bar{\psi }}$. Repeating the argument in (59), we also find

$$\begin{aligned} E_{n}({\bar{y}}) \le n e^{\mathrm{gen}}_{\mathrm{cell}} + 2n (\nu / l' + (1 - \nu )/l''){\bar{\rho }} e_{\mathrm{per}} + c \le n \big (e^{\mathrm{gen}}_{\mathrm{cell}} + {\bar{\rho }} e_{\mathrm{range}}(\mu ) \big ) + c, \end{aligned}$$

where we used $l' = \Lambda ^{-1}(\mu ')$, $l'' = \Lambda ^{-1}(\mu '')$, (57), (58), and the fact that $e_{\mathrm{range}}$ is affine on $[\mu ',\mu '']$. Likewise, as in the first part of the proof, ${\bar{y}}$ might not satisfy the boundary conditions, but we find some $\varepsilon \in (-c/n,c/n)$ such that $y:=(1+ \varepsilon ){\bar{y}} \in \mathcal {A}_n(\mu )$. Again we can bound $E^{n,\mu }_{\mathrm{min}} \le E_n(y)/(n-2) \le e^{\mathrm{gen}}_\mathrm{cell} + {\bar{\rho }}e_{\mathrm{range}}(\mu ) + c/n + c\varepsilon ^2 + c{\bar{\rho }} \varepsilon$. This concludes the proof. $\quad \square$

As announced right after Theorem 2.3, a chain involving waves with different discrete-wave periods (and wavelengths) can be energetically more favorable, even for $\mu \in M_{\mathrm{res}}$. Consequently, almost minimizers cannot be expected to be periodic, but only essentially periodic, i.e., periodic up to a small set of points, see (14). We close this section with an example in that direction and show that the upper bound can be improved in terms of the higher order error $n{\bar{\rho }}^2$. Recall (11) and (12).

#### Corollary 6.1

Consider $\mu = \Lambda ^{-1}(l) \in M_{\mathrm{res}}$ for $l \in 2\mathbb {N}\cap [l_{\mathrm{mid}}/2,l_{\mathrm{mid}}]$. Then for $\rho$ small enough the following holds:

(i) $E_{\mathrm{min}}^{n,\mu } \le e^{\mathrm{gen}}_{\mathrm{cell}} + {\bar{\rho }}e_{\mathrm{range}}(\mu ) + C_2/n - C_1{\bar{\rho }}^2,$ where $C_1,C_2>0$ only depend on $\rho$.
(ii) For $c=c(\rho )>0$ and $\varepsilon >0$ small enough there exists an almost minimizer $y\in \mathcal {A}_n(\mu )$ with
  $$\begin{aligned} \# \lbrace i_k \in \mathcal {C}(y)\,| \ \big ||y_{i_{k+1}} - y_{i_k}| - \lambda (\mu )\big | > \varepsilon \rbrace /n \ge c{\bar{\rho }}. \end{aligned}$$

#### Proof

We define the configuration ${\bar{y}}$ as in (60) with $l'' = l$, $l' = l-2$, and $\nu$ to be specified below. It then turns out that

$$\begin{aligned} |{\bar{y}}_n- {\bar{y}}_1| - (n-1)\Lambda (l) \ge - c + (n-1) \nu (\Lambda (l')- \Lambda (l'')), \end{aligned}$$

where $c=c(\rho )>0$ again accounts for boundary effects. For brevity, we write $d = \Lambda (l')- \Lambda (l'')$. Let $\varepsilon = (n-1)\Lambda (l) |{\bar{y}}_n- {\bar{y}}_1|^{-1}-1$. For *n* large enough we find $1 \le (n-1)/|{\bar{y}}_n- {\bar{y}}_1| \le 2$ by $\Lambda ([2,l_{\mathrm{mid}}]) \subset (2/3,1)$, see Lemma 4.5. Thus, we observe that $\varepsilon \le 2c/ (n-1) - d\nu$. We define $y = (1+\varepsilon ) {\bar{y}} \in \mathcal {A}_n(\mu )$. Repeating the arguments of the previous proof, we obtain

61 $$\begin{aligned} E_n(y)&\le n e^{\mathrm{gen}}_{\mathrm{cell}} + 2n (\nu / l' + (1 - \nu )/l''){\bar{\rho }} e_{\mathrm{per}} + c_1 + nc_1\varepsilon ^2 + nc_1{\bar{\rho }} \varepsilon \nonumber \\&= n \big (e^{\mathrm{gen}}_{\mathrm{cell}} + {\bar{\rho }} e_{\mathrm{range}}(\mu ) \big ) + 2n {\bar{\rho }} e_{\mathrm{per}} \nu \big (1/l' - 1/l'' \big ) + c_1 + nc_1\varepsilon ^2 + nc_1{\bar{\rho }} \varepsilon \end{aligned}$$

for $c_1=c_1(\rho ) \ge 1$. We further compute

62 $$\begin{aligned} c_1\varepsilon ^2 + c_1{\bar{\rho }}\varepsilon&\le c_1(8c^2/ (n-1)^2 + 2d^2\nu ^2) + 2cc_1 {\bar{\rho }}/(n-1) - c_1{\bar{\rho }} d \nu \nonumber \\ {}&\le c_2/n +c_2d^2\nu ^2 - c_1{\bar{\rho }} d \nu \end{aligned}$$

for a larger constant $c_2=c_2(\rho ) \ge 1$. By Lemma 4.3, (30), Lemma 4.5, and $l \in [ l_{\mathrm{mid}}/2, l_{\mathrm{mid}}]$ we find $d \ge c_3$ for a universal $c_3>0$. Then in view of $l_{\mathrm{mid}} = \lfloor 6/{\bar{\psi }} \rfloor$, $l \ge l_{\mathrm{mid}}/2$, (55), and the fact that $e_{\mathrm{per}}$ is independent of $\rho$, we derive

$$\begin{aligned} 4e_{\mathrm{per}} \big (1/l' - 1/l'' \big ) { = 8e_{\mathrm{per}} /( l(l-2)) }\le c_1d, \end{aligned}$$

provided that $\rho$ is small enough (which implies that $l_\mathrm{mid}$ is large). From (61)–(62) we deduce

$$\begin{aligned} E_n(y)&\le n \big (e^{\mathrm{gen}}_{\mathrm{cell}} + {\bar{\rho }} e_{\mathrm{range}}(\mu ) \big ) + c_1n {\bar{\rho }} d \nu /2 + c_1 +c_2 + nc_2d^2 \nu ^2 - nc_1{\bar{\rho }} d \nu \\&= n \big (e^{\mathrm{gen}}_{\mathrm{cell}} + {\bar{\rho }} e_{\mathrm{range}}(\mu ) \big ) - c_1n {\bar{\rho }} d \nu /2 + c_1 +c_2 + nc_2d^2 \nu ^2. \end{aligned}$$

An optimization of the last expression in terms of $\nu$ leads to the choice $\nu = c_1{\bar{\rho }}/(4c_2d)$ and division by $n-2$ gives (i). The configuration with $\nu = c_1{\bar{\rho }}/(4c_2d)$ also satisfies the property given in (ii), provided that *c* and $\varepsilon$ are chosen sufficiently small. $\quad \square$

### Lower bound and characterization of minimizers

We first derive the lower bound for the minimal energy (8). Afterwards, based on the lower bound estimates, we will provide the characterization of configuration with (almost) minimal energy.

#### Proof of Theorem 2.2, lower bound

Let $\mu \in M$ and consider $y \in \mathcal {A}_n(\mu )$. As before, the bonds and angles (2) are denoted by $b_i$ and $\varphi _i$, respectively. Choose $\mu ', \mu '' \in M_\mathrm{res}$ such that $(\mu ',\mu '') \cap M_{\mathrm{res}} = \emptyset$ and $\mu = \nu \mu ' + (1- \nu )\mu ''$ for some $\nu \in [0,1]$. Let $l' = \Lambda ^{-1}(\mu ')$, $l'' = \Lambda ^{-1}(\mu '') = l'+2 \in [2,l_\mathrm{mid}]$, and set $l_* = \nu l' + (1 -\nu ) l ''$. We note that $l_* = \Lambda ^{-1}(\mu )$ since $\Lambda$ is affine on $[l',l'']$.

*Outline of the proof:* In Step 1 we identify the set of *defects* consisting of particles where the cell energy deviates too much from the minimum. We will see that on the complement of the defect set the results from Sect. 5 are applicable. In this context, we partition the chain into various regions associated to even and odd discrete-wave periods, where the periods $l'$ and $l''$ will play a pivotal role. In Step 2 we estimate the length of the various parts, particularly using the concavity of the mapping $\Lambda$ (see (30)). In Step 3 we provide estimates for the energy of the chain and based on Lemma 5.2, Lemma 5.3, we derive relations between length and energy. Finally, in Step 4 we show that it is energetically convenient if the chain consists (almost) exclusively of waves with discrete-wave period $l'$ and $l''$, from which we can deduce the statement.

*Step 1: Partition of the chain.* Choose $0 < \delta \le \delta _0$ with $\delta _0$ being the minimum of the constants given in Lemma 4.9, Lemma 4.11, Lemma 5.2, and Lemma 5.3. We note that $\delta _0$ only depends on the choice of $v_2$, $v_3$, and $\rho$, but is independent of ${\bar{\rho }}$. Below in (84) and (93) we will eventually choose ${\bar{\rho }}$ sufficiently small in terms of $\delta _0$ whose choice then only depends on $v_2$, $v_3$, and $\rho$.

Define the index set of *defects* by

63 $$\begin{aligned} \mathcal {I}_{\mathrm{def}}&= \big \{ i = 2,\ldots ,n-1 \, |\ |b_{i-1} - {\bar{b}}|> \delta \, \text { or } \, |b_i - {\bar{b}}|> \delta \, \nonumber \\&\qquad \text { or } \, \min \lbrace |\varphi _i - \pi - {\bar{\psi }}|,|\varphi _i - \pi + {\bar{\psi }}| \rbrace >\delta \big \} \end{aligned}$$

with ${\bar{b}}$ and ${\bar{\psi }}$ from Theorem 2.1. We introduce the set and the labeling

64 $$\begin{aligned} \mathcal {I}_{\mathrm{def}}^* = \lbrace 1 ,n\rbrace \cup \mathcal {I}_{\mathrm{def}} = \lbrace i_1,\ldots ,i_{J} \rbrace , \ \ J \in \mathbb {N}. \end{aligned}$$

Notice that in the parts of the chain between indices $\mathcal {I}_{\mathrm{def}}^*$ we will be in the position to apply our results from Sect. 5. In particular,

65 $$\begin{aligned} \mathcal {I}_{\mathrm{wave}} = \lbrace i_j \,| \ j = 1,\ldots ,J-1, \ i_{j+1} - i_j \ge 2 \rbrace \end{aligned}$$

denotes the indices of the first particles of these parts of the chain. Similarly to (41), we let

66 $$\begin{aligned} \mathcal {I}_{\mathrm{sgn}} = \big \{ i = 3,\ldots , n-2 \,|\ i-1,i,i+1 \notin \mathcal {I}_{\mathrm{def}}, \ \varphi _{i} > \pi , \varphi _{i+1} < \pi \big \} \cup \mathcal {I}_{\mathrm{wave}}. \end{aligned}$$

We also introduce a decomposition of $\mathcal {I}_{\mathrm{sgn}}$ by (compare to (43))

67 $$\begin{aligned} \mathcal {I}^l_{\mathrm{sgn}} = \lbrace i \in \mathcal {I}_{\mathrm{sgn}} \, | \ i+k \notin \mathcal {I}_{\mathrm{sgn}} \cup \mathcal {I}_{\mathrm{def}} \text { for } k=1,\ldots ,l-1, \ i+l \in \mathcal {I}_{\mathrm{sgn}} \cup \mathcal {I}_{\mathrm{def}} \cup \lbrace n \rbrace \rbrace \end{aligned}$$

for $l \in \mathbb {N}$, $l \ge 2$. We will also use the notation

68 $$\begin{aligned} \mathcal {I}_{\mathrm{sgn},j} = \mathcal {I}_{\mathrm{sgn}} \cap [i_j,i_{j+1}-2], \ \ \ \ \ \ \mathcal {I}^l_{\mathrm{sgn},j} = \mathcal {I}^l_{\mathrm{sgn}} \cap [i_j,i_{j+1}-2] \end{aligned}$$

for $i_j \in \mathcal {I}_{\mathrm{wave}}$. As $i_{j}-1 \notin \mathcal {I}_{\mathrm{sgn}}$ for $i_j \in \mathcal {I}_{\mathrm{def}}^*$ and $i_j \notin \mathcal {I}_{\mathrm{sgn}}$ for $i_j \in \mathcal {I}_\mathrm{def}^* \setminus \mathcal {I}_{\mathrm{wave}}$, we get $\bigcup _{i_j \in \mathcal {I}_{\mathrm{wave}}} \mathcal {I}_{\mathrm{sgn},j} = \mathcal {I}_\mathrm{sgn}$ and $\bigcup _{i_j \in \mathcal {I}_{\mathrm{wave}}} \mathcal {I}^l_{\mathrm{sgn},j} = \mathcal {I}^l_{\mathrm{sgn}}$. Moreover, we introduce the number of particles related to different discrete-wave periods by

69a $$\begin{aligned}&n_{\mathrm{good}}' = \# \mathcal {I}^{l'}_{\mathrm{sgn}}l', \ \ n_{\mathrm{good}}'' = \# \mathcal {I}^{l''}_{\mathrm{sgn}} l'', \ \ n_{\mathrm{good}} = n_{\mathrm{good}}' + n_{\mathrm{good}}'', \ \ \ N_{\mathrm{good}} = \#(\mathcal {I}_{\mathrm{sgn}}^{l'}\cup \mathcal {I}_{\mathrm{sgn}}^{l''} ) \end{aligned}$$

69b $$\begin{aligned}&n_{\mathrm{odd}} = \sum _{l \in 2\mathbb {N}+1} \# \mathcal {I}^{l}_{\mathrm{sgn}}l, \ \ \ \ N_{\mathrm{odd}} = \sum _{l \in 2\mathbb {N}+1} \# \mathcal {I}^{l}_{\mathrm{sgn}} \end{aligned}$$

69c $$\begin{aligned}&n_{\mathrm{bad}} = \sum _{l \in 2\mathbb {N}, l \ne l',l''} \# \mathcal {I}^{l}_{\mathrm{sgn}}l, \ \ \ \ N_{\mathrm{bad}} = \sum _{l \in 2\mathbb {N}, l \ne l',l''} \# \mathcal {I}^{l}_{\mathrm{sgn}} \end{aligned}$$

69d $$\begin{aligned}&n_{\mathrm{def}} = \# \mathcal {I}_{\mathrm{def}}. \end{aligned}$$

We indicate the waves with discrete-wave period $l'$ and $l''$ as *good* since they are expected to appear in a configuration minimizing the energy (7), cf. Theorem 2.4. On the other hand, the other even discrete-wave periods are called *bad*. We also recall that in Sects. 4 and 5 we have seen that waves with odd discrete-wave period have to be treated in a different way. Below we will show that the numbers $n_{\mathrm{odd}}$, $n_{\mathrm{bad}}$, and $n_{\mathrm{def}}$ are negligible with respect to $n_{\mathrm{good}}$. From the definitions in (64), (65), and (67) we also get

70 $$\begin{aligned} n_{\mathrm{def}}+1 \ge \# \lbrace i \in \mathcal {I}_{\mathrm{def}}^* \, | \ i+1 \in \mathcal {I}_{\mathrm{def}}^* \rbrace&= (n-1) - \sum _{i_j \in \mathcal {I}_{\mathrm{wave}}} (i_{j+1} -i_j) \nonumber \\&= (n-1) -n_\mathrm{good} - n_{\mathrm{bad}} - n_{\mathrm{odd}}. \end{aligned}$$

Finally, we introduce the mean discrete-wave periods associated to the different parts. First, for the even discrete-wave periods we set

71 $$\begin{aligned} l_*^{\mathrm{good}} = n_{\mathrm{good}} ^{-1} \big ( n_{\mathrm{good}}' l' + n_{\mathrm{good}}'' l'' \big ), \ \ \ \ \ \ l^{\mathrm{bad}}_* = n_\mathrm{bad}^{-1} \sum _{l \in 2\mathbb {N}, l \ne l',l''} \# \mathcal {I}^{l}_\mathrm{sgn}l^2. \end{aligned}$$

On the other hand, for the odd discrete-wave periods we define

72 $$\begin{aligned} l_*^{\mathrm{odd}} = \Big (\sum _{l \in 2\mathbb {N}} r_l l\Big )^{-1} \sum _{l \in 2\mathbb {N}} r_l l^2, \end{aligned}$$

where $(r_l)_{l \in 2\mathbb {N}}$ is some admissible sequence in (44) with $\sum _{l \in 2\mathbb {N}} r_l l\Lambda (l) = \mathcal {L}\big ( (\# \mathcal {I}^l_{\mathrm{sgn}})_{l \in 2\mathbb {N}+1 }\big )$.

*Step 2: Length of the chain.* We consider the indices $\mathcal {I}_{\mathrm{sgn}}$ and estimate the length of the various contributions related to ‘good’, ‘bad’, and ‘odd’ discrete-wave periods. We start with the bad discrete-wave periods. Using the fact that $\Lambda$ is concave (see Lemma 4.5) and applying Jensen’s inequality, we deduce by (71)

$$\begin{aligned} \sum _{ l \in 2\mathbb {N}, l \ne l',l''} \# \mathcal {I}_{\mathrm{sgn}}^l \ l\Lambda (l)&\le n_{\mathrm{bad}} \Lambda \Big (n_{\mathrm{bad}}^{-1} \sum _{ l \in 2\mathbb {N}, l \ne l',l''} \# \mathcal {I}_{\mathrm{sgn}}^l \ l^2\Big ) = n_{\mathrm{bad}} \Lambda (l_*^{\mathrm{bad}})\nonumber \\&\le n_{\mathrm{bad}} \Lambda ( l_* ) + n_{\mathrm{bad}} \Lambda '(l_* )(l_*^{\mathrm{bad}} - l_*), \end{aligned}$$

where $\Lambda '(l_*)$ denotes the right derivative of $\Lambda$ at $l_*$. More precisely, if $l^{\mathrm{bad}}_* \in [l'-1/2,l''+1/2]$, the strict concavity of $\Lambda$ on $[2,l_{\mathrm{mid}}]$ (see Lemma 4.5 and Remark 4.6) imply

$$\begin{aligned} \sum _{ l \in 2\mathbb {N}, l \ne l',l''} \# \mathcal {I}_{\mathrm{sgn}}^l \ l\Lambda (l)&\le n_{\mathrm{bad}} \Lambda ( l_*^{\mathrm{bad}} ) - n_\mathrm{bad} c_\Lambda \\&\le n_{\mathrm{bad}} \Lambda ( l_* ) + n_{\mathrm{bad}} \Lambda '(l_* )(l_*^{\mathrm{bad}} - l_*) - n_{\mathrm{bad}} c_\Lambda \end{aligned}$$

for a constant $c_\Lambda =c_\Lambda (\rho )>0$. On the other hand, if $l^{\mathrm{bad}}_* \notin [l'-1/2,l''+1/2]$, using again the strict concavity of $\Lambda$ and $l_* \in [l',l'']$, we get

$$\begin{aligned} \Lambda ( l_*^{\mathrm{bad}} ) \le \Lambda (l_*) + \Lambda '(l_* )(l_*^{\mathrm{bad}} - l_*) -c_\Lambda , \end{aligned}$$

possibly passing to a smaller constant $c_\Lambda$. Summarizing, in both cases we get

73 $$\begin{aligned} \sum _{ l \in 2\mathbb {N}, l \ne l',l''} \# \mathcal {I}_{\mathrm{sgn}}^l \ l\Lambda (l) \le n_{\mathrm{bad}}\Lambda ( l_*) + n_\mathrm{bad}\Lambda '(l_* )(l_*^{\mathrm{bad}} - l_*) -n_{\mathrm{bad}}c_\Lambda . \end{aligned}$$

Likewise, again using (71) and the fact that $\Lambda$ is affine on $[l',l'']$, we get for the good discrete-wave periods that

74 $$\begin{aligned} \sum _{l =l',l''} \# \mathcal {I}_{\mathrm{sgn}}^{l} l\Lambda (l)&= n_{\mathrm{good}}' \Lambda (l') + n_{\mathrm{good}}'' \Lambda (l'') = n_{\mathrm{good}}\Lambda (l_*^{\mathrm{good}})\nonumber \\&\le n_{\mathrm{good}}\Lambda (l_*) + n_{\mathrm{good}}\Lambda '(l_*)( l_*^{\mathrm{good}} - l_*). \end{aligned}$$

We now address odd discrete-wave periods. Recalling the definition of the maximal length of odd discrete-wave periods in (44) and using (68), we derive

$$\begin{aligned} \sum _{i_j \in \mathcal {I}_{\mathrm{wave}}} \mathcal {L}\big ( (\# \mathcal {I}^l_{\mathrm{sgn},j})_{l \in 2\mathbb {N}+1 }\big ) \le \mathcal {L}\big ( (\# \mathcal {I}^l_{\mathrm{sgn}})_{l \in 2\mathbb {N}+1 }\big ). \end{aligned}$$

Recall that $\Lambda$ is concave. Then from (72), the fact that $\sum _{l \in 2\mathbb {N}} l r_l \le n_\mathrm{odd}$ (see (44) and (69b)), and Jensen’s inequality we get

75 $$\begin{aligned} \sum _{i_j \in \mathcal {I}_{\mathrm{wave}}} \mathcal {L}\big ( (\# \mathcal {I}^l_{\mathrm{sgn},j})_{l \in 2\mathbb {N}+1 }\big )&\le \sum _{l \in 2\mathbb {N}} r_l l\Lambda (l) \le \Big (\sum _{l \in 2\mathbb {N}} l r_l\Big ) \ \Lambda \Big ( \Big (\sum _{l \in 2\mathbb {N}} l r_l\Big )^{-1} \sum _{l \in 2\mathbb {N}} r_l l^2 \Big ) \nonumber \\&\le n_{\mathrm{odd}} \Lambda (l_*^\mathrm{odd}) \le n_{\mathrm{odd}}\Lambda (l_*) + n_\mathrm{odd}\Lambda '(l_*)(l_*^{\mathrm{odd}} -l_*). \end{aligned}$$

Now combining (73)–(75) and using (68) as well as $n_{\mathrm{good}} + n_{\mathrm{bad}} + n_\mathrm{odd} \le n-1$ (see (70)) we derive

76 $$\begin{aligned} L&:= (n-1)\Lambda (l_*) + n_{\mathrm{good}}\Lambda '(l_*)( l_*^{\mathrm{good}} - l_*) + n_{\mathrm{bad}}\Lambda '(l_*)( l_*^{\mathrm{bad}} - l_*) \nonumber \\&\quad + n_{\mathrm{odd}}\Lambda '(l_*)( l_*^{\mathrm{odd}} - l_*)\nonumber \\&\ge \sum _{i_j \in \mathcal {I}_{\mathrm{wave}}} \Big ( \sum _{l \in 2\mathbb {N}} \# \mathcal {I}_{\mathrm{sgn},j}^l l\Lambda (l) + \mathcal {L}\big ( (\# \mathcal {I}^l_{\mathrm{sgn},j})_{l \in 2\mathbb {N}+1 }\big ) \Big ) + n_{\mathrm{bad}} c_\Lambda . \end{aligned}$$

We close this step with an estimate about the contribution of $\mathcal {I}_{\mathrm{def}}$. Recall the definitions of $\mathcal {I}_\mathrm{def}^*$ and $\mathcal {I}_{\mathrm{wave}}$ in (64)–(65). For each $j \in \lbrace 1,\ldots , J-1\rbrace$, we define

77 $$\begin{aligned} \lambda _j = (y_{i_{j+1}} - y_{i_j}) \cdot e_1. \end{aligned}$$

In view of the boundary conditions $(y_n-y_1) \cdot e_1 = (n-1)\mu$ (see (6)) and the fact that the length of each bond is bounded by 3/2 (see (1)), we find by (70)

78 $$\begin{aligned} \Big | (n-1)\mu - \sum _{i_j \in \mathcal {I}_{\mathrm{wave}}} \lambda _j\Big | = \Big | \sum _{i\in \mathcal {I}^*_{\mathrm{def}}: \ i+1 \in \mathcal {I}_{\mathrm{def}}^* } (y_{i+1} - y_{i}) \cdot e_1\Big | \le 3/2(n_{\mathrm{def}}+1). \end{aligned}$$

*Step 3: Energy estimates.* First, recalling (7), (64)–(65) and defining $n_j = i_{j+1} - i_{j}+ 1 \ge 3$ for $i_j \in \mathcal {I}_{\mathrm{wave}}$, we get

79 $$\begin{aligned} E_n(y) = \sum _{j=2}^{J-1} E_3( (y_{i_j-1},y_{i_j} , y_{i_j+1} ) ) + \sum _{i_j \in \mathcal {I}_{\mathrm{wave}}} E_{n_j} \big ( (y_{i_j}, \ldots , y_{i_{j+1}}) \big ) - 2{\bar{\rho }} n_{\mathrm{def}}, \end{aligned}$$

where we used that, by the decomposition at each defect two longer-range contributions are neglected and $v_2 \ge -1$. We consider the first sum. In view of the fact that the cell energy $\tilde{E}_{\mathrm{cell}}$ is minimized exactly for $({\bar{b}},{\bar{b}},\pi + {\bar{\psi }})$ and $({\bar{b}},{\bar{b}},\pi - {\bar{\psi }})$ by Lemma 3.1, (63) implies for $j \in \lbrace 2 ,\ldots , J-1\rbrace$

80 $$\begin{aligned} \sum _{j=2}^{J-1} E_3( (y_{i_j-1},y_{i_j} , y_{i_j+1} ) ) = \sum _{j=2}^{J-1} E_{\mathrm{cell}}(y_{i_j-1}, y_{i_j}, y_{i_j+1} ) \ge n_{\mathrm{def}} (e_{\mathrm{cell}} + c_{\mathrm{def}}) \end{aligned}$$

for a constant $c_{\mathrm{def}}=c_{\mathrm{def}}(\delta )>0$. As $\delta$ depends only on $\rho$, also $c_{\mathrm{def}}$ depends only on $\rho$. On the other hand, if $i_j \in \mathcal {I}_{\mathrm{wave}}$, we can apply Lemma 5.3 and get

81 $$\begin{aligned} E_{n_j} \big ((y_{ i_j }, \ldots , y_{i_{j+1}}) \big )&\ge (n_j-3)e^{\mathrm{gen}}_{\mathrm{cell}} + e_{\mathrm{cell}} + (2 \# \mathcal {I}_{\mathrm{sgn},j} - 3 ) {\bar{\rho }} e_{\mathrm{per}} - n_jc_\mathrm{range} {\bar{\rho }}^2 + \frac{E_j^{\mathrm{red}}}{2}, \end{aligned}$$

where for brevity we have set $E_j^{\mathrm{red}} = E_{n_j}^\mathrm{red}\big ((y_{ i_j }, \ldots , y_{i_{j+1}}) \big ) - (n_j-2)e_\mathrm{cell}$. Here, we have also used that the set $\mathcal {I}_{\mathrm{sgn},j}$ coincides with the one considered in Sect. 5, see (41) and (68).

Our goal is to estimate the sum in (79). As a preparation, we recall that $|e_{\mathrm{cell}} - e^{\mathrm{gen}}_\mathrm{cell} | \le c{\bar{\rho }}$, as observed below (54), and we calculate

$$\begin{aligned}&\sum _{i_j \in \mathcal {I}_{\mathrm{wave}}} \big ((n_j-3)e^{\mathrm{gen}}_\mathrm{cell} + e_{\mathrm{cell}}\big ) + n_{\mathrm{def}} e_{\mathrm{cell}}&\ge \Big (\sum _{i_j \in \mathcal {I}_{\mathrm{wave}}} (n_j-2 ) + n_{\mathrm{def}} \Big ) e^{\mathrm{gen}}_{\mathrm{cell}} \\&\quad - (n_{\mathrm{def}} + \# \mathcal {I}_\mathrm{wave}) c{\bar{\rho }}. \end{aligned}$$

Recalling $n_j = i_{j+1} - i_{j}+ 1$, by an elementary computation, using (65) and (70), we find that $\sum _{i_j \in \mathcal {I}_{\mathrm{wave}}} (n_j-2) = (n-1) - \# \lbrace i \in \mathcal {I}_{\mathrm{def}}^* \, | \ i+1 \in \mathcal {I}_{\mathrm{def}}^* \rbrace - \#\mathcal {I}_{\mathrm{wave}}= n-2 - n_{\mathrm{def}}$. Thus, we obtain

82 $$\begin{aligned} \sum _{i_j \in \mathcal {I}_{\mathrm{wave}}} \big ((n_j-3)e^{\mathrm{gen}}_\mathrm{cell} + e_{\mathrm{cell}}\big ) + n_{\mathrm{def}} e_{\mathrm{cell}}&\ge (n-2)e^{\mathrm{gen}}_{\mathrm{cell}} - (2n_{\mathrm{def}} + 1) c{\bar{\rho }}, \end{aligned}$$

where we used that $\# \mathcal {I}_{\mathrm{wave}} \le n_{\mathrm{def}} +1$, see (64)–(65). Similarly, we compute

83 $$\begin{aligned} - \sum _{i_j\in \mathcal {I}_{\mathrm{wave}}} \big ( n_jc_{\mathrm{range}}{\bar{\rho }}^2 + 3 {\bar{\rho }}e_{\mathrm{per}}\big )&\ge - nc_{\mathrm{range}}{\bar{\rho }}^2 - \#\mathcal {I}_{\mathrm{wave}}(2c_{\mathrm{range}}{\bar{\rho }}^2 + 3 {\bar{\rho }}e_{\mathrm{per}}) \nonumber \\&\ge - nc_{\mathrm{range}}{\bar{\rho }}^2 - (n_{\mathrm{def}}+1)(2c_\mathrm{range}{\bar{\rho }}^2 + 3 {\bar{\rho }}e_{\mathrm{per}}). \end{aligned}$$

Now combining (79)–(83) and using $\sum _{i_j \in \mathcal {I}_{\mathrm{wave}}} \# \mathcal {I}_{\mathrm{sgn},j} =\#\mathcal {I}_{\mathrm{sgn}}$, we derive

$$\begin{aligned} E_n(y)&\ge (n-2)e^{\mathrm{gen}}_{\mathrm{cell}} + 2 \#\mathcal {I}_\mathrm{sgn}{\bar{\rho }}e_{\mathrm{per}} - nc_{\mathrm{range}}{\bar{\rho }}^2\\&\quad + n_{\mathrm{def}}(c_{\mathrm{def}} - 2 c{\bar{\rho }} -2c_{\mathrm{range}}{\bar{\rho }}^2 - 3 {\bar{\rho }}e_{\mathrm{per}} - 2{\bar{\rho }}) \nonumber \\&\ \ \ - 2c_{\mathrm{range}}{\bar{\rho }}^2 - 3 {\bar{\rho }}e_{\mathrm{per}} - c{\bar{\rho }} + \sum _{i_j \in \mathcal {I}_{\mathrm{wave}}} \frac{1}{2}E^{\mathrm{red}}_j. \end{aligned}$$

As $c_{\mathrm{def}} = c_{\mathrm{def}}(\rho )$ and $c_{\mathrm{range}} = c_\mathrm{range}(\rho )$ are independent of ${\bar{\rho }}$, we can select ${\bar{\rho }}$ so small that the last term in the first line can be bounded from below by $n_{\mathrm{def}}c_{\mathrm{def}}/2 + n_\mathrm{def}{\bar{\rho }}e_{\mathrm{per}}$. Thus, we derive

84 $$\begin{aligned} E_n(y)&\ge (n-2)e^{\mathrm{gen}}_{\mathrm{cell}} + 2 \#\mathcal {I}_\mathrm{sgn}{\bar{\rho }}e_{\mathrm{per}} - nc_{\mathrm{range}} {\bar{\rho }}^2 \nonumber \\&+ n_{\mathrm{def}}(c_{\mathrm{def}}/2 +{\bar{\rho }}e_{\mathrm{per}}) -c_\mathrm{rest}{\bar{\rho }} +  \sum _{i_j \in \mathcal {I}_\mathrm{wave}} \frac{E^{\mathrm{red}}_j}{2} \end{aligned}$$

for $c_{\mathrm{rest}} = 2c_{\mathrm{range}}{\bar{\rho }} + 3 e_{\mathrm{per}} + c$. The next steps will be to derive suitable lower bounds for $2 \#\mathcal {I}_{\mathrm{sgn}}{\bar{\rho }}e_{\mathrm{per}}$ and $\sum _{i_j \in \mathcal {I}_{\mathrm{wave}}} E^{\mathrm{red}}_j/2$ . We first estimate the latter. Recalling (68), (77), and the definition of $E_j^{\mathrm{red}}$ in (81), we find by Lemma 5.2

$$\begin{aligned} E_{j}^{\mathrm{red}} \ge \frac{c_{\mathrm{el}}}{n_j} \Big (\lambda _j - \sum _{l \in 2\mathbb {N}} \# \mathcal {I}_{\mathrm{sgn},j}^l l\Lambda (l) -\mathcal {L}\big ( (\# \mathcal {I}^l_{\mathrm{sgn},j})_{l \in 2\mathbb {N}+1 }\big ) + n^j_{\mathrm{odd}}c_{\mathrm{odd}} - { 4 } l_{\mathrm{max}} \Big )^2_+, \end{aligned}$$

where $n_{\mathrm{odd}}^j = \sum _{l \in 2\mathbb {N}+1} \# \mathcal {I}^{l}_{\mathrm{sgn},j} l$. Here, we have again used that the sets $\mathcal {I}_{\mathrm{sgn},j}$ and $\mathcal {I}_{\mathrm{sgn},j}^l$ coincide with the ones considered in Sect. 5. By a computation similar to the one before (82), using $\#\mathcal {I}_{\mathrm{wave}} \le n_{\mathrm{def}} + 1$, we get $\sum _{i_j \in \mathcal {I}_{\mathrm{wave}}} n_j = n-2 - n_{\mathrm{def}} + 2\# \mathcal {I}_{\mathrm{wave}} \le n+n_{\mathrm{def}} \le 2n$. Then, taking the sum over all $i_j \in \mathcal {I}_{\mathrm{wave}}$ and using Jensen’s inequality, we derive

$$\begin{aligned} \sum _{i_j \in \mathcal {I}_{\mathrm{wave}}} E^{\mathrm{red}}_j\ge & {} \frac{c_{\mathrm{el}}}{2n} \Big ( \sum _{i_j \in \mathcal {I}_{\mathrm{wave}}} \Big ( \lambda _j - \sum _{l \in 2\mathbb {N}} \# \mathcal {I}_{\mathrm{sgn},j}^l l\Lambda (l) \\&-\mathcal {L}\big ( (\# \mathcal {I}^l_{\mathrm{sgn},j})_{l \in 2\mathbb {N}+1 }\big ) + n^j_{\mathrm{odd}}c_{\mathrm{odd}} - 4 l_{\mathrm{max}}\Big ) \Big )^2_+. \end{aligned}$$

Consequently, in view of (76), (78), and $\#\mathcal {I}_{\mathrm{wave}} \le n_\mathrm{def} + 1$, we find

85 $$\begin{aligned}&\sum _{i_j \in \mathcal {I}_{\mathrm{wave}}} E^{\mathrm{red}}_j \nonumber \\&\ge \frac{c_{\mathrm{el}}}{2n} \Big ( (n-1)\mu - 3/2(n_{\mathrm{def}}+1) - L + n_{\mathrm{bad}} c_\Lambda + n_{\mathrm{odd}}c_{\mathrm{odd}} - 4 (n_{\mathrm{def}} +1)l_{\mathrm{max}} \Big )^2_+, \end{aligned}$$

where we have also used $\sum _{i_j \in \mathcal {I}_{\mathrm{wave}}} n_{\mathrm{odd}}^j = n_{\mathrm{odd}}$, see (68) and (69b).

We now consider the term $2 \#\mathcal {I}_{\mathrm{sgn}}{\bar{\rho }}e_\mathrm{per}$. Recall that $\Upsilon$, defined in (57), is convex. By Jensen’s inequality we compute with (69c) and (71)

86 $$\begin{aligned} \sum _{ l \in 2\mathbb {N}, l \ne l',l''} 2 \# \mathcal {I}_{\mathrm{sgn}}^l&= n_{\mathrm{bad}} \sum _{ l \in 2\mathbb {N}, l \ne l',l''} n_{\mathrm{bad}}^{-1} \ \# \mathcal {I}_{\mathrm{sgn}}^l \ l \Upsilon (l) \ge n_{\mathrm{bad}} \Upsilon \Big (n_{\mathrm{bad}}^{-1} \sum _{ l \in 2\mathbb {N}, l \ne l',l''} l^2 \# \mathcal {I}_{\mathrm{sgn }}^l \Big )\nonumber \\ {}&= n_\mathrm{bad} \Upsilon (l_*^{\mathrm{bad}})\ge n_{\mathrm{bad}} \big ( \Upsilon (l_*) + \Upsilon '(l_*)(l_*^{\mathrm{bad}} - l_*)\big ), \end{aligned}$$

where $\Upsilon '(l_*)$ denotes the right derivative of $\Upsilon$ at $l_*$. Likewise, for the good discrete-wave periods using (69a), (71), and the fact that that $\Upsilon$ is affine on $[l',l'']$ we obtain

87 $$\begin{aligned} 2 N_{\mathrm{good}}&= 2\# (\mathcal {I}^{l'}_{\mathrm{sgn}} \cup \mathcal {I}^{l''}_{\mathrm{sgn}}) = n_{\mathrm{good}}' \Upsilon (l') + n_{\mathrm{good}}'' \Upsilon (l'') = n_{\mathrm{good}} \Upsilon \big (n_{\mathrm{good}}^{-1} (n_{\mathrm{good}}' l' + n_{\mathrm{good}}'' l'') \big )\nonumber \\&= n_{\mathrm{good}} \Upsilon (l_*^{\mathrm{good}}) \ge n_{\mathrm{good}} \big ( \Upsilon (l_*) + \Upsilon '(l_*)(l_*^{\mathrm{good}} - l_*) \big ). \end{aligned}$$

Finally, using (69b), (72), and the facts that $\sum _{l \in 2\mathbb {N}} r_l = \sum _{l \in 2\mathbb {N}+1} \# \mathcal {I}^l_{\mathrm{sgn}}$, $\sum _{l \in 2\mathbb {N}} l r_l \ge n_\mathrm{odd}-1$ (see (44)) we obtain for the odd discrete-wave periods by $\Upsilon (l^{\mathrm{odd}}_*) \le 1$ and Jensen’s inequality

88 $$\begin{aligned} \sum _{ l \in 2\mathbb {N}+1} 2 \# \mathcal {I}_{\mathrm{sgn}}^l = \sum _{l \in 2\mathbb {N}} 2r_l = \sum _{l \in 2\mathbb {N}} r_l l\Upsilon (l)&\ge (n_\mathrm{odd}-1) \Upsilon (l_*^{\mathrm{odd}}) \ge n_{\mathrm{odd}} \Upsilon (l_*^\mathrm{odd}) - 1 \nonumber \\&\ge n_{\mathrm{odd}} \big ( \Upsilon (l_*) + \Upsilon '(l_*)(l_*^{\mathrm{odd}} - l_*) \big ) - 1. \end{aligned}$$

In view of (70), (84)–(88), the fact that $\mu = \Lambda (l_*)$, , and the definition of *L* in (76), we now obtain the energy estimate

89 $$\begin{aligned} E_n(y)&\ge (n-2)e^{\mathrm{gen}}_{\mathrm{cell}} + (n-2)\Upsilon (l_*){\bar{\rho }}e_{\mathrm{per}} - nc_{\mathrm{range}} {\bar{\rho }}^2 + n_{\mathrm{def}}c_{\mathrm{def}}/2 -(c_{\mathrm{rest}} + e_{\mathrm{range}}){\bar{\rho }}\nonumber \\&\quad + \Big ( n_{\mathrm{good}}\Upsilon '(l_*)(l_*^{\mathrm{good}} - l_*) + n_{\mathrm{bad}}\Upsilon '(l_*)(l_*^{\mathrm{bad}} - l_*) + n_{\mathrm{odd}}\Upsilon '(l_*)(l_*^{\mathrm{odd}} - l_*) \big ) \Big ){\bar{\rho }}e_{\mathrm{per}} \nonumber \\&\quad + \frac{c_{\mathrm{el}}}{4n} \Big ( (n_{\mathrm{odd}} + n_{\mathrm{bad}}) \min \lbrace c_{\mathrm{odd}},c_\Lambda \rbrace - 6 (n_{\mathrm{def}} +1)l_{\mathrm{max}}\nonumber \\&\quad - n_{\mathrm{good}}\Lambda '(l_*)(l_*^{\mathrm{good}} - l_*) - n_{\mathrm{bad}}\Lambda '(l_*)( l_*^{\mathrm{bad}} - l_*) - n_\mathrm{odd}\Lambda '(l_*)( l_*^{\mathrm{odd}} - l_*)\Big )^2_+. \end{aligned}$$

*Step 4: Conclusion:* We are now in the position of establishing the lower bound for the energy. We will show that

90 $$\begin{aligned} \frac{1}{n-2} E_n(y) - \big ( e^{\mathrm{gen}}_{\mathrm{cell}} + \Upsilon (l_*) {\bar{\rho }} e_{\mathrm{per}}\big ) \ge - c \Big ({\bar{\rho }}^2 + \frac{{\bar{\rho }}}{n} \Big ) \end{aligned}$$

for a constant $c=c(\rho )>0$. In view of the definition (58) and $l_* = \Lambda ^{-1}(\mu )$, this yields the claim. For brevity, we set $\beta _1 = n_{\mathrm{good}}/(n-2)$, $\beta _2 = (n_\mathrm{bad} + n_{\mathrm{odd}})/(n-2)$, $\beta _3 = n_{\mathrm{def}}/(n-2)$, and $\tilde{l} = (n_{\mathrm{bad}} l_*^{\mathrm{bad}} + n_{\mathrm{odd}} l_*^\mathrm{odd})/(n_{\mathrm{bad}} + n_{\mathrm{odd}})$. Moreover, we let

91 $$\begin{aligned} H = \big ( \beta _1 \Upsilon '(l_*)(l_*^{\mathrm{good}} - l_*) + \beta _2 \Upsilon '(l_*)(\tilde{l} - l_*)\big ) {\bar{\rho }} e_{\mathrm{per}} \ \ \ \text { and } \ \ \ \kappa = \Lambda (l_*)/(\Upsilon '(l_*) {\bar{\rho }} e_{\mathrm{per}}). \end{aligned}$$

Dividing (89) by $n-2$, we see that, in order to derive (90), it suffices to show that

92 $$\begin{aligned} \begin{aligned} G&:= \beta _3c_{\mathrm{def}}/2 + H +\frac{c_{\mathrm{el}}}{8} \Big (\beta _2 \min \lbrace c_{\mathrm{odd}},c_\Lambda \rbrace - 6 (\beta _3 +2/n)l_{\mathrm{max}} - \kappa H \Big )_+^2 \\&\ge - C({\bar{\rho }}^2 +{\bar{\rho }}/n) \end{aligned} \end{aligned}$$

for $C=C(\rho )>0$. (Without restriction, we have supposed that $n \ge 4$ such that $n-2 \ge n/2$.) To this end, we minimize the term on the right with respect to *H* and observe that the minimum is attained when *H* satisfies

$$\begin{aligned} 1- \kappa c_{\mathrm{el}}/4 \big (\beta _2 \min \lbrace c_{\mathrm{odd}},c_\Lambda \rbrace - 6 (\beta _3 +2/n)l_{\mathrm{max}} - \kappa H \big )_+ =0, \end{aligned}$$

which leads to

$$\begin{aligned} H = \beta _2 \min \lbrace c_{\mathrm{odd}},c_\Lambda \rbrace /\kappa - 4/(\kappa ^2 c_{\mathrm{el}}) - 6 (\beta _3 +2/n)l_{\mathrm{max}} /\kappa . \end{aligned}$$

Thus, we obtain

$$\begin{aligned} G \ge \beta _3c_{\mathrm{def}}/2 + \beta _2 \min \lbrace c_\mathrm{odd},c_\Lambda \rbrace /\kappa - 4/(\kappa ^2 c_{\mathrm{el}}) - { 6 } (\beta _3 +2/n)l_{\mathrm{max}} /\kappa + 2/(\kappa ^2 c_{\mathrm{el}}). \end{aligned}$$

We recall from (55) and (91) that $1/\kappa \le c{\bar{\rho }}$ for a constant $c=c(\rho )>0$. Consequently, $6 l_{\mathrm{max}} /\kappa \le c_{\mathrm{def}}/4$ when ${\bar{\rho }}$ is chosen sufficiently small. (Recall that $c_{\mathrm{def}} = c_{\mathrm{def}}(\rho )$, see (80).) Since $1/\kappa \le c{\bar{\rho }}$ and the constants $c_{\mathrm{el}}$, $l_{\mathrm{max}}$ depend only on $\rho$, this gives

93 $$\begin{aligned} G&\ge \beta _3c_{\mathrm{def}}/4 + \beta _2 \min \lbrace c_{\mathrm{odd}},c_\Lambda \rbrace /\kappa - 2/(\kappa ^2 c_{\mathrm{el}}) - 12 l_{\mathrm{max}} /(n\kappa ) \nonumber \\&\ge \beta _3c_{\mathrm{def}}/4 + \beta _2 \min \lbrace c_\mathrm{odd},c_\Lambda \rbrace /\kappa - C({\bar{\rho }}^2 +{\bar{\rho }}/n) \end{aligned}$$

for $C(\rho )> 0$. The minimum is attained for $\beta _2 = \beta _3 = 0$ and thus (92) holds. This concludes the proof. $\quad \square$

We close with the characterization of almost minimizers.

#### Proof of Theorem 2.3 and Theorem 2.4

We treat the general case $\mu \in M$ considered in Theorem 2.4, from which the proof of Theorem 2.3 can be deduced directly. Choose $\mu ', \mu '' \in M_{\mathrm{res}}$ such that $(\mu ',\mu '') \cap M_{\mathrm{res}} = \emptyset$ and $\mu = \nu \mu ' + (1- \nu )\mu ''$ for some $\nu \in [0,1]$. Let $l' = \Lambda ^{-1}(\mu ')$, $l'' = \Lambda ^{-1}(\mu '') = l'+2$ and recall from (15) that $\lambda (\mu ') = l'\Lambda (l')$ and $\lambda (\mu '') = l''\Lambda (l'')$. We also let $l_* = \nu l' + (1-\nu )l''$ and observe that $l_* = \Lambda ^{-1}(\mu )$ since $\Lambda ^{-1}$ is piecewise affine. Suppose that $n{\bar{\rho }}^2 \ge 1$. In the following, $C>0$ denotes a generic constant which may always depend on $\rho$ and $l_{\mathrm{max}}$ (and thus only on $\rho$, cf. Remark 3.2).

Suppose that $y \in \mathcal {A}_n(\mu )$ is an almost minimizer, see (12). From the proof of the lower bound of Theorem 2.2 we derive that (84) and (89) hold. In particular, we recall the notations *G*, *H*, $\beta _1$, $\beta _2$, and $\beta _3$ in (91)–(92). By (89) and (58) we get

$$\begin{aligned} \frac{1}{n-2}E_n(y)\ge & {} e^{\mathrm{gen}}_{\mathrm{cell}} + \Upsilon (l_*) {\bar{\rho }} e_{\mathrm{per}} - c \big ( {\bar{\rho }}^2+ {\bar{\rho }}/n\big ) + G \\= & {} e^{\mathrm{gen}}_{\mathrm{cell}} + {\bar{\rho }} e_{\mathrm{range}}(\mu ) - c \big ( {\bar{\rho }}^2+ {\bar{\rho }}/n\big ) + G. \end{aligned}$$

Theorem  2.2, the fact that $n{\bar{\rho }}^2 \ge 1$, and (12) imply

94 $$\begin{aligned} E_{\mathrm{min}}^{n,\mu } + c {\bar{\rho }}^2\ge \frac{1}{n-2}E_n(y) \ge E_{\mathrm{min}}^{n,\mu } - c {\bar{\rho }}^2 + G \end{aligned}$$

which together with (93) gives $\beta _3 c_\mathrm{def}/4 + \beta _2 \min \lbrace c_{\mathrm{odd}}, c_\Lambda \rbrace /\kappa \le C {\bar{\rho }}^2.$ As $\delta =\delta (\rho )$ and $\kappa \le c'/{\bar{\rho }}$ for some $c'=c'(\rho )>0$ (see (91)), this yields

95 $$\begin{aligned} (n_{\mathrm{bad}}+n_{\mathrm{odd}})/(n-2)&=\beta _2 \le C {\bar{\rho }}, \nonumber \\ \ \ \ n_{\mathrm{def}}/(n-2)&=\beta _3 \le C {\bar{\rho }}^2, \nonumber \\ \ \ n_{\mathrm{good}}/(n-2)&= \beta _1 \ge 1 - C{\bar{\rho }}. \end{aligned}$$

The last estimate follows from the fact that $\beta _1 + \beta _2 +\beta _3 \ge (n-2)/(n-2)=1$, see (70). Recall $N_{\mathrm{good}}$ from (69a) and $\mathcal {I}_{\mathrm{sgn}}$ from (66). By (69b), (69c), and (95), we deduce

96 $$\begin{aligned} (\# \mathcal {I}_{\mathrm{sgn}} - N_{\mathrm{good}}) / n_{\mathrm{good}} = (N_\mathrm{bad} + N_{\mathrm{odd}}) / n_{\mathrm{good}} \le (n_{\mathrm{bad}} + n_{\mathrm{odd}}) / n_{\mathrm{good}} \le C {\bar{\rho }}. \end{aligned}$$

From (92), (94), and (95) we get $H + (C'{\bar{\rho }}- \kappa H)_+^2 c_{\mathrm{el}}/8\le C {\bar{\rho }}^2$, where $C'=C'(\rho ) \in \mathbb {R}$. Since $1/\kappa \le c' {\bar{\rho }}$ by (91), this implies $|H| \le C {\bar{\rho }}^2$ after some computations. Recalling the definition of *H* in (91) and using (95), we find

$$\begin{aligned} |\beta _1\Upsilon '(l_*)(l_*^{\mathrm{good}} - l_*) { {\bar{\rho }}e_{\mathrm{per}} } | \le C {\bar{\rho }}^2 + |\beta _2 \Upsilon '(l_*)(\tilde{l}-l_*){\bar{\rho }}e_{\mathrm{per}}| \le C {\bar{\rho }}^2 \end{aligned}$$

where $l_*^{\mathrm{good}}$ was defined in (71). With (95) we get $|l_*^{\mathrm{good}} - l_*| \le C {\bar{\rho }}$, which by (71) and $l_* = \nu l' + (1-\nu )l''$ implies

97 $$\begin{aligned} |l_*^{\mathrm{good}} - l_*| = | ( n_{\mathrm{good}}' l' + n_{\mathrm{good}}'' l'') /n_{\mathrm{good}} - \big (\nu l' + (1-\nu )l''\big ) | \le C{\bar{\rho }}. \end{aligned}$$

Using $l'' - l' =2$ we obtain by a short computation

98 $$\begin{aligned} | n_{\mathrm{good}}'/n_{\mathrm{good}} - \nu | + |n_{\mathrm{good}}''/n_{\mathrm{good}} - (1-\nu )| \le C{\bar{\rho }}. \end{aligned}$$

We introduce the parameter $\sigma = 2\nu /(l' \Upsilon (l_*))$ appearing in (17). Note that $\sigma$ only depends on $\mu$, but is independent of *y*. Moreover, we have $1-\sigma = 2(1-\nu )/(l'' \Upsilon (l_*))$. This follows after some computations taking $\Upsilon (l_*) = (2\nu l'' + 2(1-\nu )l')/(l'l'')$ into account, where the latter is due to $l_* = \nu l' + (1-\nu )l''$ and the fact that $\Upsilon$, defined in (57), is affine on $[l',l'']$. For later purpose, we also note that by $2N_{\mathrm{good}} = n_{\mathrm{good}} \Upsilon (l_*^\mathrm{good})$ (see (87)), (96), and (97) we get

99 $$\begin{aligned} |2 \#\mathcal {I}_{\mathrm{sgn}} - n_{\mathrm{good}}\Upsilon (l_*)|&\le |2N_{\mathrm{good}} - n_{\mathrm{good}}\Upsilon (l_*)| + Cn_{\mathrm{good}}{\bar{\rho }}\nonumber \\&= n_{\mathrm{good}}| \Upsilon (l_*^{\mathrm{good}}) - \Upsilon (l_*)| + Cn_{\mathrm{good}}{\bar{\rho }} \le Cn_{\mathrm{good}}{\bar{\rho }} \end{aligned}$$

for a constant *C* depending also on the Lipschitz constant of $\Upsilon$. We now show that

100 $$\begin{aligned} | \# \mathcal {I}_{\mathrm{sgn}}^{l'} - \sigma \#\mathcal {I}_{\mathrm{sgn}}|/n + | \# \mathcal {I}_{\mathrm{sgn}}^{l''} - (1-\sigma )\#\mathcal {I}_\mathrm{sgn}|/n \le C {\bar{\rho }}. \end{aligned}$$

Indeed, by using $l' \# \mathcal {I}_{\mathrm{sgn}}^{l'}=n_{\mathrm{good}}'$, $\sigma = 2\nu /(l' \Upsilon (l_*))$, (98), and (99) we calculate

$$\begin{aligned} \frac{1}{n}| \# \mathcal {I}_{\mathrm{sgn}}^{l'} - \sigma \#\mathcal {I}_{\mathrm{sgn}}|&= \frac{n_{\mathrm{good}}}{nl'} | n_\mathrm{good}'/n_{\mathrm{good}} - \sigma \# \mathcal {I}_{\mathrm{sgn}} l'/n_\mathrm{good}| \le | n_{\mathrm{good}}'/n_{\mathrm{good}}\\&\quad - \nu | + C{\bar{\rho }} \le C{\bar{\rho }}. \end{aligned}$$

For $\# \mathcal {I}_{\mathrm{sgn}}^{l''}$ we argue likewise taking $1-\sigma = 2(1-\nu )/(l'' \Upsilon (l_*))$ into account.

Now (100) is the starting point to prove (16)–(17). We have to show that most of the waves satisfy (16). To this end, fix $\varepsilon > 0$ and recalling $l'\Lambda (l') = \lambda (\mu ')$, $l''\Lambda (l'') = \lambda (\mu '')$ we define

$$\begin{aligned} \mathcal {K}^{\mathrm{bad}} = \bigcup _{l = l',l''} \lbrace i \in \mathcal {I}_{\mathrm{sgn}}^{l} \, | \ \big ||y_{i+l}-y_i| - l\Lambda (l)\big | { > } \varepsilon \rbrace , \end{aligned}$$

as well as $\mathcal {K}' = \mathcal {I}_{\mathrm{sgn}}^{l'} \setminus \mathcal {K}^{\mathrm{bad}}$ and $\mathcal {K}'' = \mathcal {I}_\mathrm{sgn}^{l''} \setminus \mathcal {K}^{\mathrm{bad}}$. To conclude the proof of (16)–(17), it now remains to show that

101 $$\begin{aligned} \# \mathcal {K}^{\mathrm{bad}}/n \le C_\varepsilon {\bar{\rho }} \end{aligned}$$

for a constant $C_{\varepsilon }=C_\varepsilon (\varepsilon ,\rho )$ additionally depending on $\varepsilon$. Indeed, the claim follows from the definition of $\mathcal {K}^{\mathrm{bad}}$, (100), and the fact that $|\# \mathcal {C}(y) - \# \mathcal {I}_{\mathrm{sgn}}| \le Cn{\bar{\rho }}^2$ (see (11), (66), (69d), and (95)).

We now prove (101). Recalling (65), (67), and applying Lemma 4.9 we get

$$\begin{aligned} \sum _{l=l',l''}\sum _{i \in \mathcal {I}_{\mathrm{sgn}}^{l}} (|y_{i+l}-y_i| - l\Lambda (l))^2_+ \le C\sum _{i_j \in \mathcal {I}_{\mathrm{wave}}} E^{\mathrm{red}}_j \end{aligned}$$

for $C=C(\rho )$, where the abbreviation $E^{\mathrm{red}}_j$ was introduced in (81). Then, by using (84), we find

$$\begin{aligned} \sum _{l=l',l''}\sum _{i \in \mathcal {I}_{\mathrm{sgn}}^{l}} (|y_{i+l}-y_i| - l\Lambda (l))^2_+&\le C\big (E_n(y) - (n-2)e^{\mathrm{gen}}_{\mathrm{cell}} - 2 \#\mathcal {I}_{\mathrm{sgn}}{\bar{\rho }}e_{\mathrm{per}} \big ) +Cn{\bar{\rho }}^2 + C{\bar{\rho }}. \end{aligned}$$

Then by (58), (95), (99), $l_* = \Lambda ^{-1}(\mu )$, the fact that *y* is an almost minimizer (12), Theorem 2.2, and $n {\bar{\rho }}^2 \ge 1$ we derive

$$\begin{aligned} \sum _{l=l',l''}\sum _{i \in \mathcal {I}_{\mathrm{sgn}}^{l}} (|y_{i+l}-y_i| - l\Lambda (l))^2_+&\le Cn{\bar{\rho }}^2. \end{aligned}$$

By Hölder’s inequality we also derive

102 $$\begin{aligned} \sum _{l=l',l''}\sum _{i \in \mathcal {I}_{\mathrm{sgn}}^{l}} (|y_{i+l}-y_i| - l\Lambda (l))_+ \le C \sqrt{N_{\mathrm{good}}} \sqrt{n{\bar{\rho }}^2} \le Cn{\bar{\rho }}. \end{aligned}$$

In view of the boundary conditions $(y_n-y_1) \cdot e_1 = (n-1)\mu$ (see (6)) and the fact that the length of each bond is bounded by 3/2 (see (1)), we find by (70) and (95)

103 $$\begin{aligned} \sum _{l=l',l''} \sum _{i \in \mathcal {I}_{\mathrm{sgn}}^l} |y_{i+l} - y_i|&\ge (n-1)\mu - \frac{3}{2}((n-1) - n_{\mathrm{good}}) \ge (n-1)\mu \nonumber \\&\quad - \frac{3}{2}(n_{\mathrm{def}} + n_{\mathrm{odd}} + n_{\mathrm{bad}} +1) \nonumber \\&\ge (n-1) \mu - C ( n{\bar{\rho }}+ 1 ) \ge (n-1) \mu - Cn {\bar{\rho }}, \end{aligned}$$

where we again used that $n {\bar{\rho }}^2 \ge 1$. By (69a), (71), and (97) we find

104 $$\begin{aligned} \sum _{l=l',l''} \sum _{i \in \mathcal {I}_{\mathrm{sgn}}^l} l\Lambda (l) = n_{\mathrm{good}} \Lambda (l^{\mathrm{good}}_*) \le (n-1)\Lambda (l_*) + Cn {\bar{\rho }} = (n-1)\mu + Cn {\bar{\rho }} \end{aligned}$$

for a constant *C* depending on the Lipschitz constant of $\Lambda$. In the first equality we again used that $\Lambda$ is affine on $[l',l'']$. Combining (103) and (104) we get

$$\begin{aligned} - Cn {\bar{\rho }} \le \sum _{l=l',l''} \sum _{i \in \mathcal {I}_\mathrm{sgn}^l} (|y_{i+l} - y_i| - l\Lambda (l)). \end{aligned}$$

This together with (102) shows $\sum _{l=l',l''} \sum _{i \in \mathcal {I}_{\mathrm{sgn}}^l} \big ||y_{i+l} - y_i| - l\Lambda (l)\big | \le cn{\bar{\rho }}$ and yields (101). This concludes the proof of (16)–(17).

Finally, we recall that $\mu = \nu l' + (1-\nu )l''$ and $\sigma = 2\nu /\big (l' \Upsilon (\nu l' + (1-\nu )l'') \big )$. Thus, in case $\nu =1$ we have $\sigma = 1$ and in case $\nu = 0$ we have $\sigma = 0$. Consequently, also the special case described in Theorem 2.3 follows. $\quad \square$
